# Nanoprinted high-neuron-density optical linear perceptrons performing near-infrared inference on a CMOS chip

**DOI:** 10.1038/s41377-021-00483-z

**Published:** 2021-03-03

**Authors:** Elena Goi, Xi Chen, Qiming Zhang, Benjamin P. Cumming, Steffen Schoenhardt, Haitao Luan, Min Gu

**Affiliations:** 1 Centre for Artificial-Intelligence Nanophotonics, School of Optical-Electrical and Computer Engineering, University of Shanghai for Science and Technology, Shanghai, 200093 China; 2 Laboratory for Artificial-Intelligence Nanophotonics, School of Science, RMIT University, Melbourne, VIC 3001 Australia

**Keywords:** Optical materials and structures, Integrated optics, Lithography

## Abstract

Optical machine learning has emerged as an important research area that, by leveraging the advantages inherent to optical signals, such as parallelism and high speed, paves the way for a future where optical hardware can process data at the speed of light. In this work, we present such optical devices for data processing in the form of single-layer nanoscale holographic perceptrons trained to perform optical inference tasks. We experimentally show the functionality of these passive optical devices in the example of decryptors trained to perform optical inference of single or whole classes of keys through symmetric and asymmetric decryption. The decryptors, designed for operation in the near-infrared region, are nanoprinted on complementary metal-oxide–semiconductor chips by galvo-dithered two-photon nanolithography with axial nanostepping of 10 nm^1^^,^^2^, achieving a neuron density of >500 million neurons per square centimetre. This power-efficient commixture of machine learning and on-chip integration may have a transformative impact on optical decryption^3^, sensing^4^, medical diagnostics^5^ and computing^6^^,^^7^.

## Introduction

Communication technology is a cornerstone of modern society, making the secure exchange of information more important than ever. This demand to preserve the privacy of information, systems and networks^8,9^ has led to the development of rigid authentication schemes, which require a specific decryption key, and flexible authentication schemes using a multitude of keys. While in large-scale communication systems, data are transferred through optical signals, decryption is mostly performed in the electronic domain, requiring costly conversion of the information. Executing cryptography directly in the optical domain offers several advantages inherent to optical signals, such as propagation at the speed of light, direct information processing in two-dimensional space and parallelism. With this motivation, considerable effort has been devoted to optical security schemes through the use of phase masks^10–13^, which can be used as physical encryption and decryption keys. These phase masks are usually designed by optimisation algorithms, such as the Gerchberg–Saxton iteration^10–12^ or wavefront matching^13^. The resulting optically enabled encryption/decryption systems require multiple passes through different sets of bulky phase masks and lenses to encrypt and retrieve a message. Moreover, with their rigid constraints on inputs and keys, phase mask-based systems fail to meet the requirements for flexible authentication schemes, as used in biometric security.

**Fig. 1:**
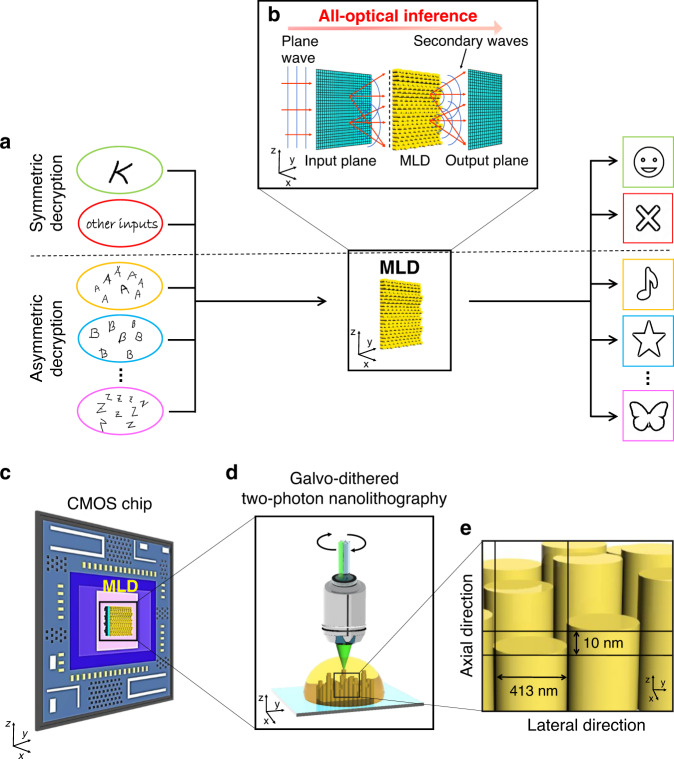
All-optical machine learning decryptor for integration on CMOS. **a** Through computer machine learning training, the optical machine learning decryptor (MLD) acquires the capabilities of identifying a single decryption key (symmetric decryption, top) or entire classes of decryption keys (asymmetric decryption, bottom), and decoding a multitude of messages using a single decryptor element. **b** The decryption system can be considered a diffractive neural network for optical inference. Each layer of the network consists of *N* × *N* artificial neurons, secondary sources of waves (details in Supplementary Methods). **c** Schematic of an MLD integrated with a CMOS chip. The nanoscale MLD is physically 3D printed by GD-TPN (**d**), a nanofabrication method that gives precise control over the MLD neuron dimensions in the lateral and axial directions (**e**), achieving axial nanostepping of 10 nm

By employing machine learning methods in optical cryptographic protocols, the limitations faced in traditional bulky optical security schemes^10–13^ can be overcome, paving the way for a new generation of compact optically enabled machine learning decryption systems for enhanced authentication solutions. Through computer-based machine learning training, the decryptors learn the ability to decode a multitude of messages and map them into a desired output, thus acquiring the capability of selectively recognising one specific decryption key among an infinite number of input keys for symmetric decryption or identifying the class to which a specific input key belongs for asymmetric decryption (Fig. 1a). Once computer-based training is completed, the decryptors can be physically fabricated as single-layer holographic perceptrons (Fig. 1b) able to recognise several input keys through all-optical machine learning inference and display the corresponding decrypted message or a notification of rejection (Table 1).

**Table 1:** Abbreviations.

MLD	Machine learning decryptor	MLD-T	MLD trained to recognise the correct key and to visually communicate the acceptance with a tick
CMLP	Compact multilayer perceptron	MLD-B	MLD trained to act as a secure display, showing the image of a butterfly
9-MLD	MLD able to decrypt nine classes of handwritten letters	MLD-T_IPS_	MLD-T optimised for IPS photoresist
3-MLD	MLD able to decrypt three classes of handwritten letters	MLD-B_IPS_	MLD-B optimised for IPS photoresist

List of acronyms and abbreviations used in the text

The single-layer perceptrons optically implement matrix multiplications^14^. Implementation of matrix multiplication in the optical domain has been a topic of research for decades^15^, and has been shown in free space through the use of beam splitters or Mach–Zehnder interferometers^16,17^, as well as in integrated photonic circuits^18,19^ through the same mechanisms, for application in optical signal processing^20^ and reconfigurable optical neural networks^18^. Recently, diffractive neural network architectures have been proposed^21^, in which these matrix multiplications are performed by diffractive elements. This marked the beginning of optical data processing through diffractive neural network inference, although the fabrication methods applied are only suitable for devices operating with a low neuron density. To utilise the full potential of diffractive machine learning networks at near-infrared (NIR) telecommunication wavelengths, it is essential to develop fabrication protocols that enable much more compact designs with optimised neuron densities (Fig. S1). Nanolithographic methods^22–27^ are an excellent candidate to fulfil this design requirement, as they can—unlike PolyJet 3D printing—precisely realise optical elements with nanometre feature sizes. Among the nanolithographic methods, galvo-dithered two-photon nanolithography (GD-TPN)^8^ stands out as the only method that allows direct fabrication of three-dimensional (3D), free-form structures in a single fabrication step with lateral and azimuthal resolution sufficient for devices, with applications in the NIR and visible wavelength regimes. In addition to the high resolution and design freedom, GD-TPN provides the flexibility to print on arbitrary substrates without concern for charged particle irradiation of off-the-shelf optoelectronic devices, such as complementary metal-oxide–semiconductor (CMOS) imaging sensors—fast, energy efficient and low-cost microelectronic circuits widely used in modern consumer products. The integration of optical machine learning decryption systems with CMOS imaging can enable unpowered optical decryption at the speed of light, with the advantage that the decrypted images can be directly transmitted, displayed and stored over standard electronic communication channels.

In this work, we present a novel concept for compact optical decryptors that can be integrated on common CMOS chips (Fig. 1c–e). Using computer machine learning based on error back-propagation methods, single-layer holographic perceptrons are trained to perform critical decryption of single or whole classes of images. By nanoprinting the machine learning decryptors (MLDs), which are designed for operation in the NIR wavelength region, with GD-TPN, we achieve a neuron density of over 500 million neurons per square centimetre, while controlling the neuron height with a precision down to 10 nm.

The ability of the MLDs to execute the optical inference tasks and perform unpowered decryption of several messages at the speed of light with a working distance as small as 62.8 μm—an advantage for on-chip integration—is experimentally shown. By printing the MLDs directly on a CMOS chip, we achieve compact and highly integrated devices, which not only outperform current optical decryption methods, but also show the potential for application of full optical inference devices in a wide range of fields from computer vision to medical diagnostics.

## Results

### Design, training and optimisation

The MLD presented in this article is a single diffractive element capable of scattering and directionally focusing each of a multitude of images given as input and of mapping them into a specific output. Once printed, the MLD can optically perform the inference tasks of a single-layer perceptron, mapping a variety of images on a sensor, effectively realising the functionalities of decryption.

#### Computer-based machine learning training

The compact decryption system can be considered a diffractive neural network^21,28^ working in transmission mode. We modelled the MLD system on a computer to perform the training. In our model, the neural network is composed of three layers (input, MLD and output), each consisting of *N* × *N* resolvable pixels that act as artificial neurons, which receive, modulate and transmit a light field (Fig. 1b). The neurons of each layer are linked to the neurons of the neighbouring layers through Rayleigh–Sommerfeld^29^ diffraction. While the neurons of the input and output layers are unbiased (i.e., uniform), each neuron of the diffractive layer adds a bias in the form of a phase delay to the transmitted signal. A cross-entropy loss function is defined to evaluate the performance of the MLD with respect to the desired target, and a machine learning algorithm iteratively optimises the phase delay of each neuron in the diffractive layer to minimise the loss function (Fig. S2). The ‘Methods’ and Supplementary Materials sections contain the details of this TensorFlow-based design and training processes.

#### Compact multilayer training

**Fig. 2:**
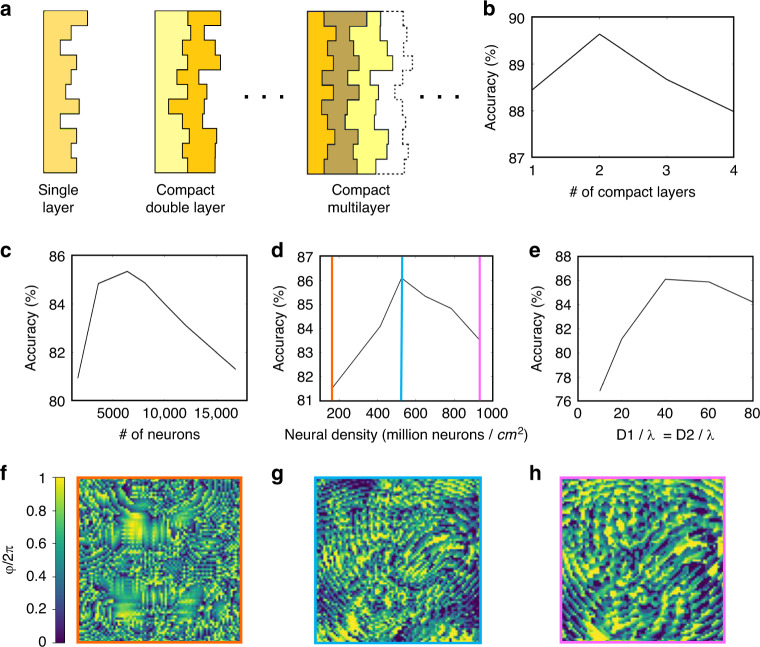
Neuron density effect on the MLD optical inference ability. **a** Schematic of MLD designs consisting of one, two and multiple compact layers. **b** Blind test classification accuracy of the 9-MLD as a function of the number of compact layers. The pixel diameter is 419 nm, and the distances from the input plane to the compact layers and from the compact layer to the output plane are 70.7 and 31.4 μm, respectively. Classification accuracy achieved by the 9-MLD as a function of the number of neurons (**c**), neuron density (**d**), and distance from the input plane to the MLD (D1) and from the MLD to the output plane (D2) (**e**). **f**–**h** Calculated phase delay in the diffractive layer with the neuron density values as indicated by the coloured markers in **d**, showing the effect of the neuron density on the MLD design

The MLD perceptron^30^ is a basic neural network building block that is shallow and can only learn linearly separable functions. In a system composed of multiple diffractive layers with a sufficient physical separation between them, the artificial neurons of neighbouring layers are linked through Rayleigh–Sommerfeld diffraction^31^ and can optically execute the function they are trained for. For these systems, increasing the number of layers always improves the classification accuracy (Fig. S3)^32^. The introduction of multiple diffractive layers separated in space does, however, come at the cost of losing compactness. To create a more powerful mechanism for learning that still achieves compactness, we investigate the use of a compact multilayer perceptron (CMLP; Fig. 2a), where the layers adjoin. However, unlike in the case of multiple well-separated diffractive layers, we find that an increase in the number of layers in a CMLP does not generally lead to an improvement in classification accuracy. This outcome implies that the operation implemented through multiple compact layers can be combined into a single matrix operation, which can be called a tailored linear multiplexor. The results in Fig. 2b show that a CMLP composed of two adjoining layers achieves an improvement in classification accuracy compared to a single-layer MLD.

#### Symmetric and asymmetric decryption

To demonstrate the functionalities that MLDs can achieve, we implement decryption using a specific key or classes of keys, achieving symmetric and asymmetric decryption, respectively (Fig. 1a, and Figs. S4 and S5). In symmetric or single-key cryptography, data can be encrypted and decrypted using a specific decryption key^3^ to selectively display a message. In our optical implementation of a symmetric decryption scheme, the decryption key (an image of the letter A) is the only key that, if propagated through the decryptor, retrieves the message. On the other hand, asymmetric cryptography provides security using classes of keys during the encryption process^3^. In this way, any key belonging to the key class can decrypt the corresponding message. In our optical machine learning implementation of asymmetric decryption, any key belonging to a specific key class (e.g., any image of a handwritten letter A) can decrypt the message assigned to this key class. This ability can be applied in multi-authentication schemes, such as biometric security, given that it can recognise images of the same subject under different conditions.

#### Decryptor design

To evaluate the ability of MLDs to perform symmetric decryption, we design two distinct optical decryptors. The first decryptor, MLD-T, is trained to recognise the correct key against other random keys belonging to three different classes of handwritten letters, and to visually communicate the acceptance or rejection of the input key (Figs. S6a and S7a). The second decryptor, MLD-B, acts as a secure display, showing the image of a butterfly in the output plane only if the correct input key is given. Other input keys are diffracted to the edge, leaving the output layer dark (Figs. S6b and S7b). The ability to perform asymmetric decryption is evaluated through the design of two MLDs able to decrypt nine (9-MLD) and three (3-MLD) classes of handwritten letters (Figs. S6c,d and S7c, d). Each class of input letters is decrypted into a distinct rectangular indicator on the output plane. All the decryptors are designed to operate at a wavelength of 785 nm, which was selected to match the transmission characteristics of the photoresist used during fabrication. The details of the training and test datasets are contained in the ‘Methods’ and Supplementary Materials sections.

The performance of the MLD, which is evaluated through numerical testing, is strongly influenced by the task the MLD is trained for (Fig. S8) and by the decryptor physical parameters. This is due to the impact that the size and density of the neurons have on the diffraction, and therefore on the connection between the neurons in neighbouring layers. The number of pixels (Fig. 2c and Fig. S9), neuron density (Fig. 2d and Fig. S10) and distance from the input plane to the MLD (D1) and from the MLD to the output plane (D2; Fig. 2e and Fig. S11) must therefore be finely tuned and optimised. The ‘Methods’ and Supplementary Materials sections contain the details of this optimisation process.

### MLD nanoprinting

The MLDs are realised by converting the calculated phase delay of each neuron in the diffractive layer into a relative height map (Fig. S12), that is, 3D nanoprinted using the GD-TPN method^1,2^ (Fig. S13) in hybrid zinc oxide photoresist (Fig. S14). Table ST1 and the ‘Methods’ and Supplementary Materials sections contain the details of the GD-TPN method.

**Fig. 3:**
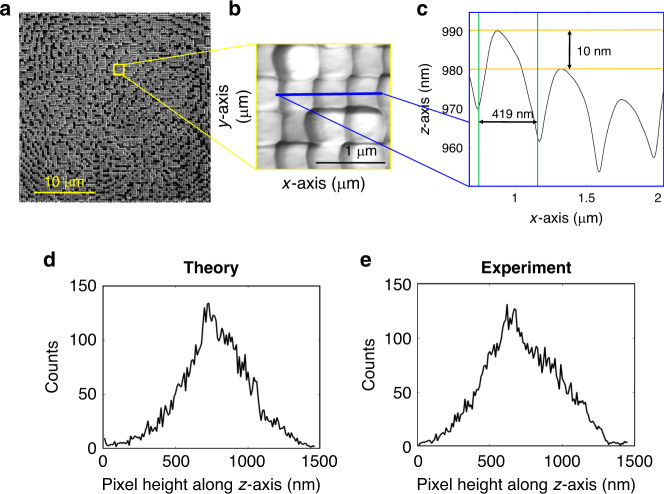
MLD nanoprinted with GD-TPN. **a** Scanning electron microscopy (SEM) image of the 3D nanoprinted 9-MLD. The two calculated compact layers (Fig. 2a) are combined in a single compact double layer and printed in a single printing session. AFM topographical image (**b**) of a 9-MLD section. **c** Height profile along a row of neurons of the 3D-printed 9-MLD. The line profile is taken along the line highlighted in blue in **b**. The yellow lines in **c** mark a step in the *z* direction of 10 nm, while the green lines mark the width of the neuron. Theoretical (**d**) and experimental (**e**) height distributions for the 3-MLD

The use of GD-TPN allows us to precisely fabricate neurons with an arbitrary diameter in the range of 200−1000 nm (ref. ^33^), which results in a maximum neuron density in the diffractive layer of 2.5 billion neurons per square centimetre. For the particular MLDs considered in this work, the optimal neuron diameter was determined to be 413 and 419 nm, resulting in a neuron density of over 500 million neurons per square centimetre. This is six orders of magnitude higher than the neuron density of current diffractive neural networks^21^. At the same time, the use of galvo-dithering correction combined with an acousto-optic modulator and a precise piezoelectric nanotranslation stage gives us control over the axial position of the focal spot with a precision down to 10 nm, therefore, allowing precise regulation of the phase modulation in the diffractive layer (Fig. 3). Images of the 3D-printed designs are shown in Fig. 3a and Fig. S15. To demonstrate the ability of the GD-TPN method to print high-quality MLDs, the neuron size and height are characterised using atomic force microscopy (AFM; Fig. 3b–e and Fig. S16). The AFM measurements clearly demonstrate that the pixel size (419 nm for 9-MLD and 413 nm for 3-MLD) and the height modulation (1.78 µm for 9-MLD and 1.48 µm for 3-MLD) of the printed MLDs are as designed through the learning process.

### Full optical inference

To characterise the optical inference ability and quantify the performance of the MLDs, we use the characterisation setup depicted in Fig. S18. The input images of the handwritten letters are generated by spatially modulating the light from a 785 nm laser source using a spatial light modulator (SLM) and projected on the input plane of the MLD, using two 4f systems. The output plane of the MLD is imaged through a lens system and detected using a charge-coupled device (CCD) camera (Fig. S17, ‘Methods’ and Supplementary Materials).

**Fig. 4:**
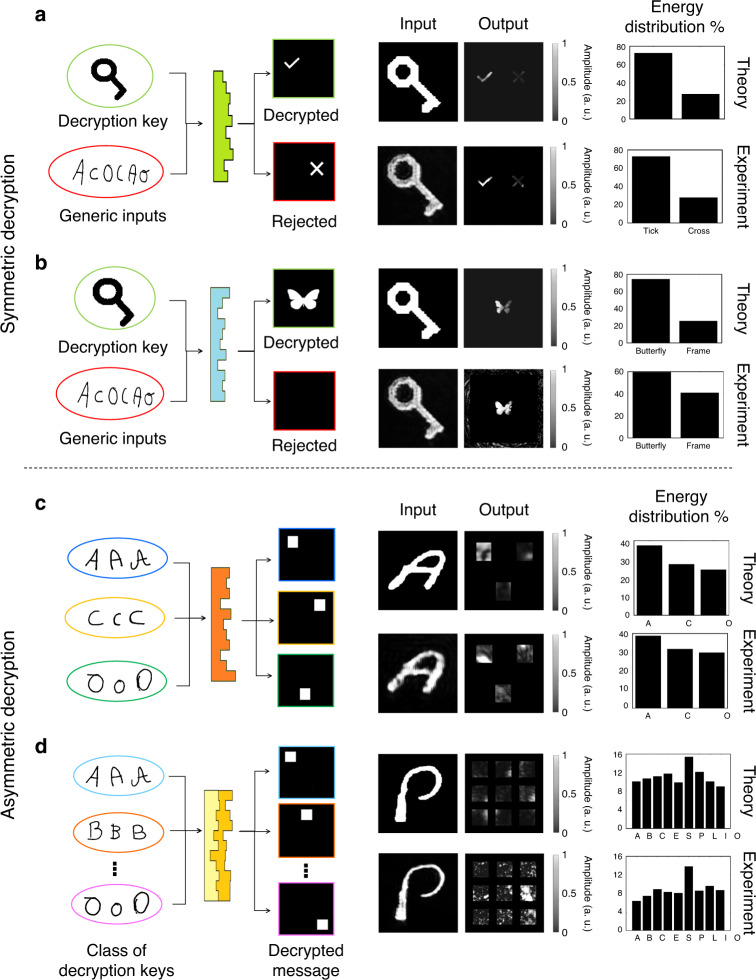
Experimental verification of the MLD performance through optical inference. Schematic of the machine learning decryption function implemented, examples of the theoretical and experimental input fields, and corresponding output patterns and energy distribution percentages for MLD-T (**a**), MLD-B (**b**), 3-MLD (**c**) and 9-MLD (**d**). The complete results and performance analysis are reported in Figs. S19–S23. The images of the input and output fields consist of 236 × 236 pixels (1.3 × 1.3 mm^2^) in the case of 3-MLD, 274 × 274 pixels (1.5 × 1.5 mm^2^) in the case of 9-MLD and 340 × 340 pixels (1.88 × 1.88 mm^2^) in the case of MLD-T and MLD-B. Each MLD output image was multiplied with the mask related to that specific MLD design before quantifying the intensity distribution (see Supplementary Materials)

To measure the experimental classification accuracy, we compare the numerical and experimental output of the MLD for five different images per letter class. In Fig. 4a, b, and Figs. S18 and S19, we report the characterisation of symmetric decryptors, MLD-T and MLD-B, as shown in Fig. S6a, b. The experimental results quantitatively match the theoretical expectation of 100% accuracy, proving that the GD-TPN nanoprinted MLDs can act as reliable symmetric decryptors and secure displays. For the asymmetric 3-MLD and 9-MLD decryptors, the results in Fig. 4c, d and Figs. S20–S22 clearly show the ability to direct the input images to the detector region assigned to the corresponding letter class. To further evaluate the performance of the 3D-printed MLD and understand the role of noise in our experimental results, we calculate the diffraction efficiencies (see Table ST2 and Supplementary Methods) and the accuracy of our MLDs, with varying degrees of normalised noise added to the camera readout (Fig. S23). The experimental diffraction results are thereby comparable with the results reported in the literature for single-layer diffractive neural networks^32^. The match between the experimental and numerical accuracies is found to be 86.67% for the 3-MLD and 80% for the 9-MLD. While the output pattern in Fig. 4d is grainier than that in Fig. 4c due to the more complex diffractive element pattern, the diminished performances of the experimental MLDs compared with the numerical results can be explained by 3D printing errors, unaccounted absorption-related losses due to the inhomogeneous material and other experimental error sources in the characterisation setup.

### CMOS integration

Recently, photonics has been leveraging on-chip technology to cope with the growing demand for optical communications in networking and industrial applications. To deploy our new principle in an on-chip application, we print MLDs on CMOS chips. Compared with other technologies, such as CCD sensors, CMOS chips are faster, more energy efficient, cheaper and already widely used in modern consumer products. The combination of all-optical MLDs with CMOS technology can enable harnessing of their complementary physics through integrated solutions on a single chip^34^, meeting the demand for a large bandwidth combined with low-energy consumption and cost (Supplementary Movie S1).

**Fig. 5:**
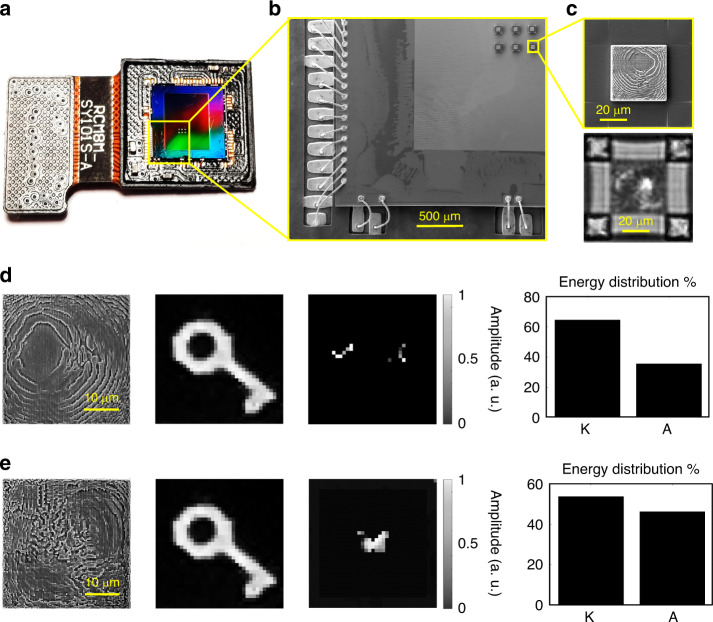
Machine learning integrated on CMOS. **a** Photograph of the Sony IMX219 NoIR CMOS sensor with an array of 3 × 2 MLDs 3D printed via the GD-TPN method. **b** SEM image of the CMOS sensor surface with the MLD array. **c** Single MLD built on the CMOS sensor imaged with SEM (top) and directly with the CMOS sensor (bottom, scale bar 50 µm = 45 pixels). SEM image of the MLD diffractive layer, example of the experimental input field, and corresponding output pattern and energy distribution percentage for the MLD-T_IPS_ (**d**) and MLD-B_IPS_ (**e**) designs. The complete results and performance analysis are reported in Figs. S30 and S31. The images of the input and output fields consist of 37 × 37 pixels (41.9 × 41.9 µm^2^)

We demonstrate the direct manufacturing and imaging of MLDs on a CMOS sensor (Fig. 5a–c and Fig. S23). For GD-TPN fabrication, we use a dip-in approach^35^ and a liquid photoresist as opposed to the zirconium-based photoresist used in the previous experiments. This is due to the zirconium-based photoresist deposition and development methods being incompatible with fabrication on the packaged CMOS chip. To ensure the proper distance between the MLD and the CMOS chip surface, we printed the MLDs on pillars. Further information on the pretreatments, design and nanoprinting can be found in Figs. S24 and S25, and the ‘Methods’ and Supplementary Materials sections.

To show the mechanical stability and repeatability of printing MLDs on a CMOS chip, we fabricate an array of MLDs (Fig. S24). The SEM (Fig. 5 and Fig. S27) and AFM (Fig. S28) characterisations prove that MLDs with the required geometry can successfully be printed on CMOS chips with the GD-TPN method. The images acquired by the CMOS sensor reported in Fig. 5d,e and Fig. S29 confirm the optical quality of the MLDs and their ability to decrypt the key image, with an accuracy of 100%. Compared with the performance of MLDs printed using hybrid zinc oxide photoresist, this experiment yields a poorer match between the experimental and numerical test results in terms of the energy distribution and intensity contrast for the *tick* and *cross* output in the case of MLD-T printed with IPS (MLD-T_IPS_), and for the *butterfly* and *frame* output in the case of MLD-B printed with IPS (MLD-B_IPS_; Fig. S30). These results can be explained by unaccounted optical losses, structural distortions due to material shrinkage, asymmetries in the pixel shapes and lower resolution images, all factors that affect the quality of the output image.

## Discussion

In this work, we have presented high-neuron-density MLDs for optical decryption through all-optical inference in the NIR wavelength region. We realise compact and highly integrated decryptors by nanoprinting the MLDs directly on a CMOS chip, using GD-TPN. Our experimental results demonstrate the application of MLDs as power-efficient optical decryptors and secure functional displays. By combining unpowered, pretrained smart optical devices with optical imaging sensors, we enable the sensors to perform complex functions as simply as putting glasses on them.

The nanoscale neuron size within the NIR MLDs not only provides the advantage of a high neuron density, but also results in a short distance (the MLD operative distance, i.e., the distance between the input and output planes, is one to three orders of magnitude smaller than that in other implementations^16,18,19,21^) and more connections between the neurons due to the increased diffraction angles. These features lead to a three orders of magnitude increase in the operational frequency, and thus in the operations per second (FLOPS) compared with the devices in the THz region (see Table ST3 and Supplementary Methods). In this regard, with superresolution^36^ and chemical etching^37^ methods, smaller feature sizes can be achieved (<10 nm), potentially creating a completely new platform for smart holographic machine learning systems.

The performance of the presented decryptors has to be critically evaluated with respect to the intended application. The security the decryptors presented in this work can provide is limited by the number of key classes they are trained to recognise, which results in a theoretical false match rate, i.e., the probability that a generic input is interpreted as a correct key, of 33% and 11% for the MLD-3 and MLD-9 decryptors, respectively.

As a machine-learning-based classification device, the decryptors presented in this work will always show a certain false match rate—a challenge inherent to the field of machine learning classification^38–40^. A number of techniques have therefore been developed to decrease the false match rate in a given classification setting, which can be equally applied to the decryptors presented in this work. For example, the training dataset can be increased to include generic inputs or random keys, which are then mapped to either the frame of the output plane or a rejection detector, as shown for MLD-B or MLD-T, respectively. In addition, a classification threshold can be applied to the output plane, in which the intensity of a given detector needs to be at a certain level above the intensity of the other detectors to be classified, as the correct decryption key. In addition, the cointegration of our MLDs directly on CMOS chips opens the possibility of further analysis of the output image collected at the detector plane in the electronic domain, which has been shown to be an energy-efficient method of hybrid optoelectronic image classification^41,42^, achieving accuracies up to 98.71% (ref. ^39^).

Our approach is based on static elements realised with linear materials. Dynamicity and optical non-linearities are elements essential for the in situ training of optical neural networks^18,43^. While reconfigurability can be incorporated into MLDs using compact reconfigurable optical elements^44–48^ and metamaterials^49–51^, non-linear materials, e.g., chalcogenide glasses^32^ or ferroelectric thin films^33^, can be used to include non-linearities, thus enabling closed-loop machine learning with the equivalent of a non-linear activation function to further improve the MLD performance^52^. The wavelength region targeted by our MLDs, the compactness and the possibility of performing a multitude of tasks, combined with the intrinsic compatibility with electronic chip manufacturing, including but not limited to CMOS chips, pave the way for a completely new generation of fast and power-efficient functional optical elements to be applied in security schemes^8,9^, medical diagnostics^5^ and computing^7,52–55^ offering a smaller footprint, a lower-energy consumption^14^ and a lower cost than present solutions.

## Materials and methods

### TensorFlow simulations

We achieve the MLD design using the TensorFlow (Google Inc.)^56^ framework, used to implement a forward propagation model, as illustrated in Fig. S2. For the free space propagation of light between different planes of the system, we employ the Rayleigh–Sommerfeld diffraction theory in the far-field regime^29^. To build a realistic model and match the experimental conditions, we consider the absorption of the material in the calculations (see Supplementary Materials) and the circular shape of the pixels. The refractive indexes and extinction coefficients are confirmed by ellipsometry (Fig. S14). We use the cross-entropy against the target image as a loss function^32^, with the aim of maximising the normalised signal of each target’s corresponding detector region, while minimising the total signal outside of all the detector regions. We employ the stochastic gradient descent algorithm Adam^57^ to back-propagate^58^ the errors and update the MLD phase parameters to minimise the loss function. The desired mapping functions between the input and output planes are achieved after ten epochs. The model is implemented using Python version 3.5.0 and TensorFlow framework version 1.4.0 (Google Inc.).

### Training dataset processing

The handwritten letter images are taken from the ‘A–Z Handwritten Alphabets’ dataset available on www.kaggle.com (ref. ^59^), which combines the NIST^60^ and MNIST^61^ datasets. The butterfly and key images are designed by us. For each letter, we use 6000 images for training, and 1000 images are used for blind testing. All the images are converted into greyscale and resized to match our designs.

### Sample nanoprinting

Polymeric^62^ MLDs are printed by the GD-TPN^1,2^ method (Fig. S12), a method based on femtosecond laser pulses and two-photon absorption. A femtosecond fibre laser (Coherent Fidelity II) combined with a frequency doubler (APE HarmoniXX) provides laser light at a wavelength of 535 nm. The laser pulses with a width of 55 fs and a repetition rate of 70 MHz are steered by a combination of a two-dimensional galvo mirror (Thorlabs), and a 4f imaging system into a 1.4 NA 100× oil immersion objective (Olympus). Compared with the classic TPN, the circular motion of the mirrors exposes a larger lateral volume of material while simultaneously reducing the total exposure in the axial direction and improving the axial resolution^1^. A piezoelectric nanotranslation stage (Physik Instrumente) is used to trace out the microstructures in the photoresist, while the galvo mirrors trace the laser focus in a circle. A zirconium-based hybrid organic–inorganic photoresist is used to create the templates due to its excellent resistance to shrinkage^62^. After the GD-TPN procedure, the sample is rinsed in a 1-propanol:2-propanol (30:70) solvent mixture for 30 min and then dried at room temperature.

We manufacture MLDs on a Sony IMX219 NoIR CMOS image sensor from a Raspberry Pi Camera Module. We develop a dip-in GD-TPN approach, using commercial IPS (Nanoscribe GmbH) photoresist. Before manufacturing, we remove the microlenses and clean the sensor surface with isopropanol. To precisely regulate the distance between the structures and the imaging plane, the MLDs are mounted on supports with a height of 47.1 µm. After the GD-TPN procedure, the sample is developed in SU-8 developer, rinsed with isopropanol and then dried at room temperature.

### IR testing setup

A schematic diagram of the experimental setup is given in Fig. S17. The light beam is generated through a Thorlabs OBIS 785 nm laser source. The polarised beam is directed on a Hamamatsu SLM X13138-07 (620–1100 nm). After this, two 4f systems resize the image of the handwritten letter to match the MLD dimensions and focus it on the input imaging plane. The use of a high-magnification objective (Olympus UPLANFL N, 60× 0.9 NA) in the 4f system is necessary to obtain an input image, with a size compatible with the MLD. After passing through the MLD, the signal is collected by an objective (Olympus UPLANFL N, 60× 0.9 NA), focused on the output plane and detected by a CCD camera (Basler ace acA2040-90uc, frame rate 90 Hz). In the case of the MLDs printed on the CMOS sensor, the output image is collected directly by the CMOS sensor (Sony IMX219 NoIR, frame rate 60 Hz).

## Supplementary information

Supplementary materials

Supplementary movie 1

## Data Availability

The data that support the results within this paper and other findings of the study are available from the corresponding authors upon reasonable request.

## Code availability

The custom code and mathematical algorithm used to obtain the results within this paper are available from the corresponding authors upon reasonable request.

## References

[1] Turner MD (2013). Miniature chiral beamsplitter based on gyroid photonic crystals. Nat. Photonics.

[2] Goi E, Cumming BP, Gu M (2018). Impact of cubic symmetry on optical activity of dielectric 8-srs networks. Appl. Sci..

[3] Muniraj, I. & Sheridan, J. T. *Optical Encryption and Decryption* (SPIE, 2019).

[4] Watts S (2010). Optical microchip sensors. Nat. Photonics.

[5] Kononenko I (2001). Machine learning for medical diagnosis: history, state of the art and perspective. Artif. Intell. Med..

[6] Solli DR (2003). Photonic crystal polarizers and polarizing beam splitters. J. Appl. Phys..

[7] Brunner D (2013). Parallel photonic information processing at gigabyte per second data rates using transient states. Nat. Commun..

[8] Kolata GB (1977). Computer encryption and the national security agency connection. Science.

[9] Cho A (2019). Codemakers find algorithms immune to quantum hacks. Science.

[10] Deng SG (2006). Hiding an image in cascaded Fresnel digital holograms. Chin. Opt. Lett..

[11] Shi YS (2006). Optical image hiding in the Fresnel domain. J. Opt. A Pure Appl. Opt..

[12] Liu ZJ (2010). Image watermarking by using phase retrieval algorithm in gyrator transform domain. Opt. Commun..

[13] Fontaine, N. K., Ryf, R., Chen, H., Neilson D., & Carpenter, J. Design of high order mode-multiplexers using multiplane light conversion. In *Proceedings of 2017 European Conference on Optical Communication*, 1–3 (IEEE, Gothenburg, Sweden, 2017) 10.1109/ECOC.2017.8346129.

[14] Wetzstein G (2020). Inference in artificial intelligence with deep optics and photonics. Nature.

[15] Leith EN (2000). The evolution of information optics. IEEE J. Sel. Top. Quantum Electron..

[16] Reck M (1994). Experimental realization of any discrete unitary operator. Phys. Rev. Lett..

[17] del Hougne P, Lerosey G (2018). Leveraging chaos for wave-based analog computation: demonstration with indoor wireless communication signals. Phys. Rev. X.

[18] Shen YC (2017). Deep learning with coherent nanophotonic circuits. Nat. Photonics.

[19] Ibeiro A (2016). Demonstration of a 4 × 4-port universal linear circuit. Optica.

[20] Scholtz, A. V. Optical matrix processing: a review. In *Proceedings of COMSIG 88@m_Southern African Conference on Communications and Signal Processing*, 109–114 (IEEE, Pretoria, South Africa, 1988) 10.1109/COMSIG.1988.49312.

[21] Lin X (2018). All-optical machine learning using diffractive deep neural networks. Science.

[22] Blanco A (2000). Large-scale synthesis of a silicon photonic crystal with a complete three-dimensional bandgap near 1.5 micrometres. Nature.

[23] Subramania G (2010). Log-pile TiO_2_ photonic crystal for light control at near-UV and visible wavelengths. Adv. Mater..

[24] Gissibl T (2016). Two-photon direct laser writing of ultracompact multi-lens objectives. Nat. Photonics.

[25] Sun, H. B. & Kawata, S. in *NMR • 3D Analysis • Photopolymerization* (eds Fatkullin, N. et al.) 169–273 (Springer, 2004).

[26] Fuechsle M (2012). A single-atom transistor. Nat. Nanotechnol..

[27] Eigler DM, Schweizer EK (1990). Positioning single atoms with a scanning tunnelling microscope. Nature.

[28] Zhang QM (2019). Artificial neural networks enabled by nanophotonics. Light.: Sci. Appl..

[29] Goodman, J. W. *Introduction to Fourier Optics*, 3rd edn (Roberts & Co., 2005).

[30] Rosenblatt, F. *The Perceptron: a Perceiving and Recognizing Automaton* (Cornell Aeronautical Laboratory, 1957).

[31] LeCun Y, Bengio Y, Hinton G (2015). Deep learning. Nature.

[32] Mengu D (2020). Analysis of diffractive optical neural networks and their integration with electronic neural networks. IEEE J. Sel. Top. Quantum Electron..

[33] Goi E, Cumming BP, Gu M (2018). Gyroid “srs” networks: photonic materials beyond nature. Adv. Opt. Mater..

[34] Pospischil A (2013). CMOS-compatible graphene photodetector covering all optical communication bands. Nat. Photonics.

[35] Bückmann T (2012). Tailored 3D mechanical metamaterials made by dip-in direct-laser-writing optical lithography. Adv. Mater..

[36] Gan ZS (2013). Three-dimensional deep sub-diffraction optical beam lithography with 9 nm feature size. Nat. Commun..

[37] Ramanan V (2008). Three dimensional silicon-air photonic crystals with controlled defects using interference lithography. Appl. Phys. Lett..

[38] Barreno M (2010). The security of machine learning. Mach. Learn..

[39] Singh S, Prasad SVAV (2018). Techniques and challenges of face recognition: a critical review. Procedia Comput. Sci..

[40] Adeshina YO, Deeds EJ, Karanicolas J (2020). Machine learning classification can reduce false positives in structure-based virtual screening. Proc. Natl Acad. Sci. USA.

[41] Chang JL (2018). Hybrid optical-electronic convolutional neural networks with optimized diffractive optics for image classification. Sci. Rep..

[42] Mengu, D., Luo, Y., Rivenson, Y., & Ozcan, A. Integration of diffractive optical neural networks with electronic neural networks (Conference Presentation). In *Conference on Lasers and Electro-Optics, OSA Technical Digest* (Optical Society of America, 2020), paper STh4M.2. 10.1109/JSTQE.2019.2921376 PMC7677864 33223801

[43] Zhou TZ (2020). In situ optical backpropagation training of diffractive optical neural networks. Photonics Res..

[44] Arbabi E (2018). MEMS-tunable dielectric metasurface lens. Nat. Commun..

[45] Almohammadi H, Bagnani M, Mezzenga R (2020). Flow-induced order–order transitions in amyloid fibril liquid crystalline tactoids. Nat. Commun..

[46] Li SQ (2019). Phase-only transmissive spatial light modulator based on tunable dielectric metasurface. Science.

[47] Liu XL, Padilla WJ (2013). Dynamic manipulation of infrared radiation with mems metamaterials. Adv. Opt. Mater..

[48] Roy T (2018). Dynamic metasurface lens based on MEMS technology. APL Photonics.

[49] Shanei MM (2019). All-silicon reconfigurable metasurfaces for multifunction and tunable performance at optical frequencies based on glide symmetry. Sci. Rep..

[50] Wang Q (2016). Optically reconfigurable metasurfaces and photonic devices based on phase change materials. Nat. Photonics.

[51] He Q, Sun SL, Zhou L (2019). Tunable/reconfigurable metasurfaces: physics and applications. Research.

[52] Goi E (2020). Perspective on photonic memristive neuromorphic computing. PhotoniX.

[53] Solli DR, Jalali B (2015). Analog optical computing. Nat. Photonics.

[54] Hermans M (2015). Trainable hardware for dynamical computing using error backpropagation through physical media. Nat. Commun..

[55] Papaioannou M (2016). Two-dimensional control of light with light on metasurfaces. Light. Sci. Appl..

[56] TensorFlow. Large-scale machine learning on heterogeneous systems. https://tensorflow.google.cn/ (2015).

[57] Kingma, D. P. & Ba, J. Adam: a method for stochastic optimization. In *Proceedings of the 3rd International Conference on Learning Representations* (ICLR, San Diego, USA, 2015).

[58] Rumelhart DE, Hinton GE, Williams RJ (1986). Learning representations by back-propagating errors. Nature.

[59] Kaggle. A-Z handwritten alphabets in. csv format. https://www.kaggle.com/sachinpatel21/az-handwritten-alphabets-in-csv-format/metadata (2018).

[60] Grother, P. J. NIST special database 19: NIST handprinted forms and characters database www.nist.gov/srd/nist-special-database-19 (2016).

[61] Lecun Y (1998). Gradient-based learning applied to document recognition. Proc. IEEE.

[62] Terzaki K (2011). 3D conducting nanostructures fabricated using direct laser writing. Opt. Mater. Express.

