# Supplementary material for: Nanoprinted high-neuron-density optical linear perceptrons performing near-infrared inference on a CMOS chip
Source: Light Sci Appl. 2021 Mar 3;10:40. doi: 10.1038/s41377-021-00483-z (PMC7925536; doi:10.1038/s41377-021-00483-z)
Supplement: Supplementary file 1 — Supplementary materials [file 41377_2021_483_MOESM1_ESM.docx]

Supplementary Materials

for

**Nanoprinted high-neuron-density optical linear perceptrons performing near-infrared inference on a CMOS chip**

Elena Goi^1,2^, Xi Chen^1^, Qiming Zhang^1^, Benjamin P. Cumming^2^, Steffen Schoenhardt^1^, Haitao Luan^1^, Min Gu^1,2*^

^1^Centre for Artificial-Intelligence Nanophotonics, School of Optical-Electrical and Computer Engineering,

University of Shanghai for Science and Technology, Shanghai, 200093, China.

^2^Laboratory for Artificial-Intelligence Nanophotonics, School of Science, RMIT University, Melbourne, 3001, Australia.

*Corresponding author: [gumin@usst.edu.cn](mailto:gumin@usst.edu.cn)

**Supplementary Methods**

**Wave analysis**

The machine learning decryptor (MLD) can be considered a system composed by three holographic layers, each consisting of $N\times N$ resolvable pixels that act as artificial neurons able to receive, modulate and transmit a light field. Each neuron can be considered as a source of secondary waves with amplitude and phase determined by the product of the input field and the transmission coefficients of each neuron, as illustrated in **Figure 1b**. The first layer under coherent illumination generates an input represented by a $N\times N$ complex-valued vector. The second layer (the diffractive layer) can modulate amplitude or phase of the incoming image, while the output layer is an image collected by a detector. The neurons of each layer are linked to the neurons of the neighbouring layer through free-space wave propagation following the Rayleigh-Sommerfeld^1^ diffraction equation in the far field regime:

$w_{i}\left( x,y,z \right)=\frac{z- z_{i}}{r^{2}} \left( \frac{1}{2\pi r}+ \frac{1}{j\lambda} \right)e^{\left( \frac{j2\pi r}{\lambda} \right)}$*,* (Eq. 1)

where *i* represents the *i*-th pixel of a given layer of the system located at $(x_{i},y_{i},z_{i})$ position, 𝜆 is the operative wavelength $r= \sqrt{{(x- x_{i})}^{2}+{(y- y_{i})}^{2}+ {(z- z_{i})}^{2}}$ and $j= \sqrt{-1}$. The product of the input wave and the *i*-th pixel transmission coefficient (*b_i_*) determine the amplitude and relative phase of this secondary wave. Based on this, at the output layer of the MLD, the output function $y_{{out}_{i}}\left( x,y,z \right)$of the i-th pixel located at $(x_{i},y_{i},z_{i})$ position can be written as^2^:

$y_{{out}_{i}}$ $\left( x,y,z \right)= w_{i}\left( x,y,z \right) \cdot b_{i}(x_{i},y_{i},z_{i}) \cdot y_{{in}_{i}}(x_{i},y_{i},z_{i})$*,* (Eq. 2)

where:

$y_{{in}_{i}}(x_{i},y_{i},z_{i}) = \sum_{k} {(w}_{k}\left( x,y,z \right) \cdot y_{0_{k}}\left( x_{i},y_{i},z_{i} \right))$, (Eq. 3)

$b_{i}(x_{i},y_{i},z_{i}) = a_{i}(x_{i},y_{i},z_{i}) e^{\left( j \Phi_{i}(x_{i},y_{i},z_{i}) \right)}$, (Eq. 4)

$a_{i}$ $(x_{i},y_{i},z_{i})$ = ${a_{i}}_{0}\left( x_{i},y_{i},z_{i} \right)\cdot e^{\left( -\alpha\cdot z \right)}$. (Eq. 5)

$y_{0_{i}}\left( x,y,z \right)$is the field distribution at the input field, ${a_{i}}_{0}(x_{i},y_{i},z_{i})$ is the amplitude coefficient (in our case is constant and equal to 1), α = $\frac{4\pi\kappa}{\lambda}$ is the absorption coefficient of the MLD, $\kappa$ is the extinction coefficient of the MLD material, z = $\frac{\Phi}{2\pi}$ ∙ $\frac{\lambda}{\Delta n}$, λ is the wavelength, *Δn* is the difference between the refractive index of the MLD material and air, and Φ is the phase value of each MLD pixel.

The MLD can work in reflection or in transmission mode, and both phase and amplitude values of each neuron can be adjusted, providing a complex wave modulation. In our work we consider only the case of coherent transmissive MLDs and phase only modulation. Since we consider the absorption of the photoresist ($\kappa=0.02$ for the zirconium-based photoresist^3^, and $\kappa=0$ for IP‐S (by Nanoscribe GmbH), each pixel modulates also the amplitude of the incoming field, but this parameter is related to the phase modulation through **Eq. 5** and is not an independent variable.

**TensorFlow-based design and training**

As an analogy to perceptrons^4^, the transmission coefficient of each neuron can be considered as a learnable parameter that can be iteratively adjusted during the computer-based training, implementing an error back-propagation method (**Figure S2**).

We obtained the MLDs design using TensorFlow (Google Inc.) framework. The forward propagation model and its corresponding TensorFlow implementation is illustrated in **Figure S2** and can be summarised by equations **Eq. 2** – **Eq. 5**. The amplitude of the input field $y_{in}\left( x,y,z \right)$carries the information related to the input key. Each neuron of the diffractive layer adds a bias in form of a phase delay to the transmitted signal to map each input key into a specific output pattern $y_{out}\left( x,y,z \right)$. The bias of each neuron is adjusted during the training. The calculated output intensity distribution and the target output intensity distribution were used to calculate the cross-entropy loss function during the training. Since minimizing the cross-entropy loss function is equivalent to maximizing the similarity between the calculated and the target output intensity distribution, this loss function is in general more suitable for classification tasks than mean squared error loss. We used the stochastic gradient descent algorithm, Adam^5^, to back-propagate the errors and update the MLD to minimize the loss function. Note that, cross-entropy acts on probability measures, which take values in the interval (0,1) and the signals coming from the detectors (one for each class) at the output of the MLD are not necessarily in this range; therefore, in the training phase, a softmax layer is introduced to enable the use the cross-entropy loss. Although softmax is used during the training process, once the diffractive design converges and is fixed, the class assignment at the output plane of the MLD is still based solely on the maximum optical signal detected at the output plane in correspondence of the target output^6^. At the end of the numerical training, all the phase delay of the neurons are optimised, and the design is fixed. The MLD can be physically fabricated to perform the tasks for which it was trained for.

**Parameter optimisation**

We studied how different physical parameters affect the performance of the MLDs (**Figure S8-S11**). Fixing parameters such as the number of pixels (3600), size and shape of pixels (circular pixels with diameter 392.5, λ/2), and distances from input and diffractive layer (D1) and from diffractive layer to output plane (D2) to 31.4 μm, we observe that the accuracy achievable linearly depends on the number of object classes that we use as encryption keys (**Figure S8**). We study how different physical parameters affect the performance of the MLDs and we conclude that the pixel number (**Figure S9**), the neuron density (**Figure S10**), D1 and D2 (**Figure S11**) must be adjusted according to the number of encrypted object classes.

The optimised 9-MLD consists of two compact layers (phase modulation of each layer 0 - 0.6∙2π) of 6400 pixels each, with a diameter of 419 µm, D1 = 70.7 μm and D2 = 31.4 μm (**Figure S6c** and **S7c**). For the 3-MLD we fix the number of pixels to be 4900 and we optimise the design, achieving a blind test classification accuracy of 97.87% (**Figure S6d** and **S7d**). To decrypt three classes of objects the ideal number of pixels is 1600. However, we opted for a larger number of pixels to facilitate the characterization process. The final 3-MLD design consists of a single layer (phase modulation 0-2π) of 4900 pixels with a diameter of 413 µm, D1 = D2 = 55.0 μm.

The optimised designs of both MLD-T and MLD-B consist of a single layer (phase modulation 0-2π) of 10000 pixels with a diameter of 413 µm, D1 = D2 = 47.1 μm (**Figure S6a, S6b, S7a** and **S7b**). The operative wavelength λ was 785 nm and the absorption coefficient α was 0.02. The systems were trained for 10 epochs with a training dataset of 18000 images in the case of 3-MLD, 54000 images in the case of 9-MLD, and 24000 images in the case of MLD-T and MLD-B. We trained the network on circular shaped pixels to match the experimental conditions.

The MLD encryption was implemented using Python version 3.5.0. and TensorFlow framework version 1.4.0 (Google Inc.). Using a desktop computer (Helwlett-Packard HP Z640 Workstation, processor Intel(R) Xeon(R) CPU E5-2620 v4 @ 2.10GHz and 64GB of RAM, running a Windows 7 operating system, Microsoft), the TensorFlow based designs of the MLDs took approximately 1 hour to train the 3-MLD, 2 hours for the 9-MLD, and 6 hours for the MLD-T, MLD-B, MLD-T_IPS_ and the MLD-B_IPS_.

**Galvo-Dithered two-photon nanolithography**

*Galvo-Dithered two-photon nanolithography setup*

TPN is a 3D nano-fabrication technique developed over the past two decades to what is now a well-established technology, with several commercially available systems on the market^7^. The MLD were fabricated using a custom-made Galvo-Dithered two-photon nanolithography (GD-TPN)^8,9^ system (**Figure S13a**).

A femtosecond fibre laser (Coherent Fidelity II) combined with a frequency doubler (APE harmonixx) provides laser light at a wavelength of 535 nm. The laser pulses with a width of 55 fs and a repetition rate of 70 MHz, and it is steered by a 4f imaging system into a 1.4 NA × 100 oil immersion objective (Olympus). An acousto-optic modulator is used to control the light exposure during fabrication and also acts as a noise filter, reducing power fluctuations from the laser. A piezoelectric nano-translation stage (P-545.xR8S PInano® XY(Z) Piezo System Physik Instrumente) mounted on a stepper motor controller (Thorlabs, Newton, NJ, USA) was used to trace out the MLD in a photoresist. The build volume of the piezoelectric nano-translational stage is 200 × 200 × 200 μm^3^, while the build volume of the stepper motor is 25 × 25 × 25 mm^3^.

The most important addition to the GD-TPN setup is the introduction of two computer-controlled galvo-mirrors (Thorlabs), electromechanical instruments that deflect a light beam with a mirror on receipt of an electronic signal. Using galvo-mirrors, the dithering of the focal spot is achieved. The mirrors trace out a circular path in the focal plane whose radius (R) is comparable to the voxel resolution, at very high speeds compared to the speed of the translation stage during the GD-TPN process. For our MLD fabrications, a frequency of 1500 Hz was used, which is sufficiently fast for the translation stage speeds of 25 μm/s used. To explain the effect of the galvo dithering on the fabrication voxel, in **Figure 13b** we plot a schematic of the fabrication voxel of the original TPN and dithered GD-TPN methods, considering an a focal spot with a 3D Gaussian distribution. The dithered voxel is the result of the average of Gaussian focal spots that have been translated in a circular distribution over a period. By comparing the two fabrication voxels, we see that the fabrication voxel is widened in the focal plane (i.e. x-axis and y-axis). Most importantly, the galvo-dithering causes the fabrication voxel to become shorter in the Z direction (i.e. z-axsis), improving the overall resolution of the 3D fabrication method. Note this is a very simplistic model that does not take into account the highly complex nonlinear photo-polymerisation process that includes thermal effects and diffusion. However, it provides us with a simple and effective understanding of how galvo-dithering affects the fabrication voxel.

In our case, the use of the GD leads to the fabrication of more stable pillars with a tuneable thickness. In particular, the stability of the fabrication voxel achieved through the use of galvo-dithering correction combined with an acousto-optic modulator and a precise piezoelectric nano-translation stage, gives us control over the axial position of the focal spot with a precision down to 10 nm.

*MLD printing*

The 3D-printed MLD were obtained by converting the calculated phase value of each pixel (Φ) into a relative height map ΔZ = λΦ / 2πΔn, where Δn is the refractive index difference between the photoresist and air, and λ is the wavelength (**Figure S12** and **S14**). The 3D model of the MLDs to be 3D-printed were obtained using a Matlab code able to generate a point by point coordinate system. We print MLDs with operative wavelengths in the NIR using the GD-TPN method described above. For our designs, the use of galvo-mirrors enables the fabrication of circular pixels with a regular and symmetric shape. Moreover, it reduces the threshold power, and, in this way, it is possible to avoid fractures along the phase plates (**Figure S12f** and **S12g**).

The A zirconium-based hybrid organic–inorganic negative photoresist was used to create the templates due to its excellent resistance to shrinkage.^3^ The samples were prepared by drop-casting onto 0.13 – 0.17 mm thick cover glasses, and the resultant films were dried on a hotplate at 75 °C for 30 minutes before the photopolymerization. The heating process led to the condensation of the alkoxide groups and the formation of the inorganic matrix^3^. Next, the organic moieties attached to the inorganic backbone were polymerized using GD-TPN, resulting in the formation of irreversible and fully saturated aliphatic C-C covalent bonds. After the nanolithography process, the samples were rinsed with a solvent for 60 minutes in a 30:70 solution of 1-propanol:isopropanol, and then dried at room temperature. This procedure removes the unwritten material, leaving behind only regions where the focal spot has traced through and caused photopolymerisation to occur. The details of the fabrication parameters used for the nanoprinting of the MLDs can be found in **Table 1**.

We printed MLDs on CMOS sensors using a dip-in approach^10^ and a liquid photoresist (IPS, Nanoscribe) as opposed to the zirconium-based photoresist used in the previous experiments. This is due to the zirconium-based photoresist deposition and development methods being incompatible with fabrication on the packaged CMOS chip. As an image sensor we use a Sony IMX219 NoIR CMOS sensor from a Raspberry Pi Camera Module. Before GD-TPN, we removed the micro-lenses provided by the manufacturer (**Figure S26a**) and clean the sensor surface with isopropanol. At 785 nm wavelength, the absorption of IP-S photoresist can be neglected, therefore we compute new designs for MLD-T and MLD-B (**Figure S24-S26**) considering the material transparent, MLD-T_IPS_ and MLD-B_IPS_. The samples were prepared by drop-casting IPS directly on the CMOS surface. Next, the photoresist was photopolymerised using GD-TPN. After the nanolithography process, the samples were developed for 30 minutes in SU8 developer and then dried at room temperature. The details of the fabrication parameters used for the nanoprinting of the MLDs on CMOS chip can be found in **Table ST1**.

**Image post-processing**

All the images used to characterise a specific MLD design are collected from the same MLD and in the same exposure and gain conditions. Comparing the images collected by the Basler ace acA2040-90uc CCD camera (sensor area of 2040 pixels × 2046 pixels, pixel size 5.5 × 5.5 µm², imaging wavelength region 400-900 nm) and by the Sony IMX219 NoIR CMOS sensor (sensor area of 3280 pixels × 2464 pixels, pixel size 1.12 × 1.12 µm², imaging wavelength region 700-1000 nm), it is important to notice that, even if the CMOS sensor has a higher resolution than the CCD sensor, the output images of the MLDs were magnified ~ 45 times by a 4f system before being collected by the CCD, and therefore possessed higher resolution.

Each MLD output image was multiplied with the mask relative to that specific MLD design, before quantifying the intensity distribution. Each mask was resized to match the pixel size of the MLD output image. All post-processing was performed with MATLAB ver. R2018b.

In our work the mask was be applied in the digital post-processing phase, but it is important to note that it can also be physically implemented optically on the MLD, by placing a mask-shaped layer of non-transmissive material at the output plane.

**Computational power**

The neuron size affects the neuron density, the distance and the connections between the neurons in neighbouring layers. The size and density of the neurons have a strong impact on the diffraction and therefore on the connection between the neurons in neighbouring layers. All these features have repercussions on the operational frequency and thus on the operations per second (OPS).

Assuming the diffractive network has *m* layers separated by a distance *d*, each layer contains *N × N* neurons and the network is fully connected, than, the operational frequency and the number of operations per second (FLOPS)^11^ of our system would be:

$operational frequency=c / (d \cdot(m+1))$, (Eq. 6)

$FLOPS=2m \cdot{(N\cdot N)}^{2}\cdot operational frequency,$ (Eq. 7)

where *c* is the speed of light.

In **Table ST2** are reported the geometrical and operational parameters, the operational frequencies and the FLOPS for diffractive neural networks operating in the THz, NIR and visible wavelength regions, calculated using Eq. 6 and Eq. 7.

In addition to the operational frequency and the computational power, it is necessary to consider the speed of modulation or detection of the signal in order to evaluate the speed of the decryptor in a system. In general, the computational speed is limited by the photodetection rate. The frame rates of the detectors chosen in our experiments are: 90 Hz for the acA2040-90uc - Basler ace CCD camera, and 60 Hz for the Sony CMOS sensor. To speed up the total computation rate, it is possible to use detectors with higher speeds. In literature, detectors with a detection rates exceeding 100 GHz have been reported^12^.

**Power consumption**

The power consumption of an optical neural network during computation is given by the optical power necessary to maintain the phase modulation settings, the power loss in the circuit and the power necessary to trigger an optical nonlinearity and achieve a sufficiently high signal-to-noise ratio (SNR) at the photodetectors^13^.

In our implementation, maintaining the phase modulator settings requires no power.

Neglecting the propagation loss through the MLD and the power requirements for conversion between the electrical and optical domain, the power consumption of an MLD is mainly the amount of optical power required to support an optical nonlinearity that could be part of future implementations of the devices. If we assume a saturable absorber threshold of *p* ≃ 1 MW cm^–2^ (e.g. dyes, semiconductors and graphene) and an area of a neuron A = 0.4 $\cdot$ 0.4 μm^2^, the total power needed for forward propagation is estimated to be P = *p* $\cdot$ A $\cdot N^{2} \simeq N^{2} \cdot1.6 mW$.

Therefore, the power consumption of the MLD will be:

FLOPS/P = $m \cdot N^{2}\cdot operational frequency$ 1.5 10^3^ *operations / J* (Eq. 7)

**Diffraction efficiency**

The experimental diffraction efficiency can be calculated as the ration between the optical intensity measured over the expected detector area (I_d_) and the optical intensity of the input image (I_i_)^6^. The diffraction efficiency values reported in **Table ST2** were computed as the ratio of the mean values of I_d_ and I_i_ for the test samples that were correctly classified by the corresponding MLD designs.

**Supplementary Figures**

**
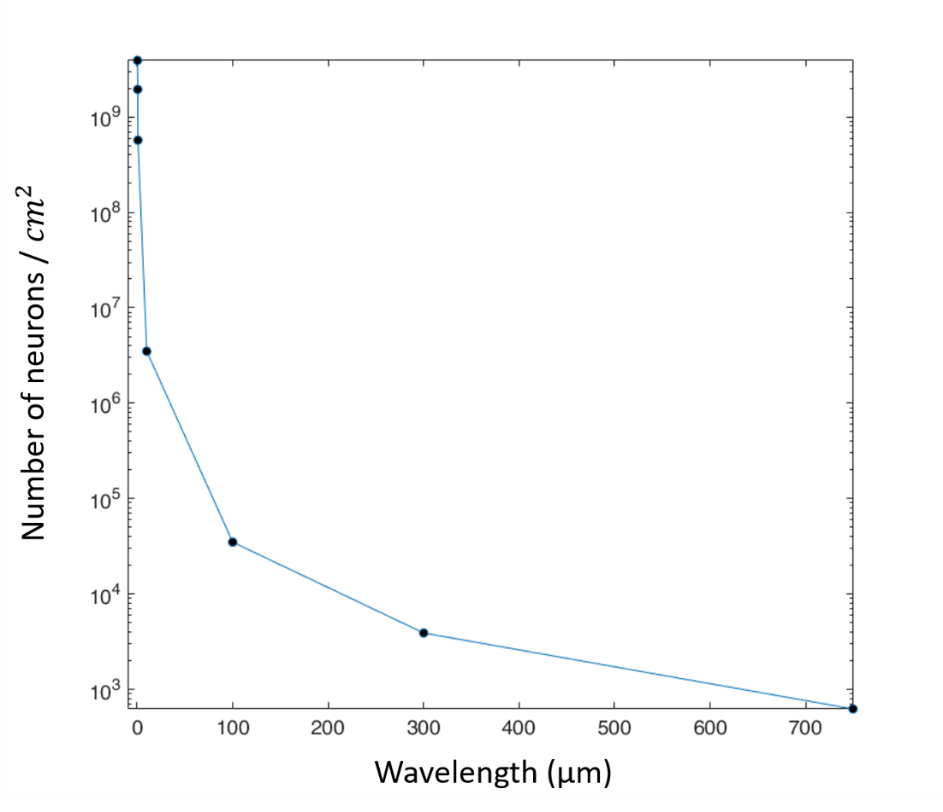
**

**Figure S1**. Number of neurons per cm^2^ (logarithmic scale) as a function of the wavelength, for an MLD performing digit classification^2^. The ration between the wavelength and the pixel diameter is 1.874^2^.

**
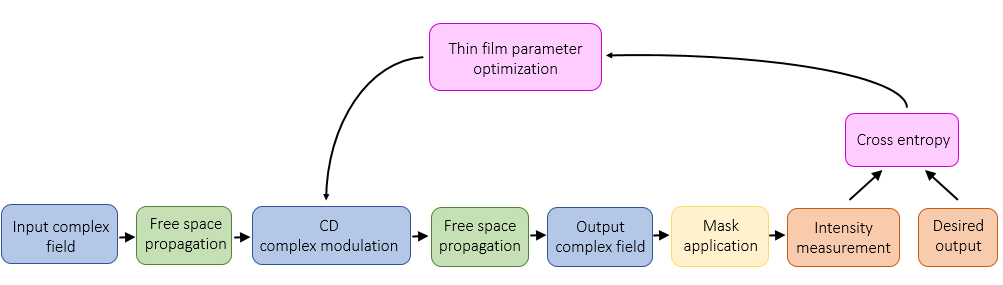
**

**Figure S2**. Schematic of a TensorFlow implementation of optical encryption^2^. After propagating in free space, the input complex field goes through the MLD that performs complex modulation. At the output plane, after free space propagation, a mask is applied to the out-coming complex field, and the intensity distribution is measured and compared with the image to encrypt. A cross entropy loss function is defined to evaluate the performance of the MLD with respect to the desired target, and the algorithm iteratively optimises the MLD parameters to minimize the loss function.


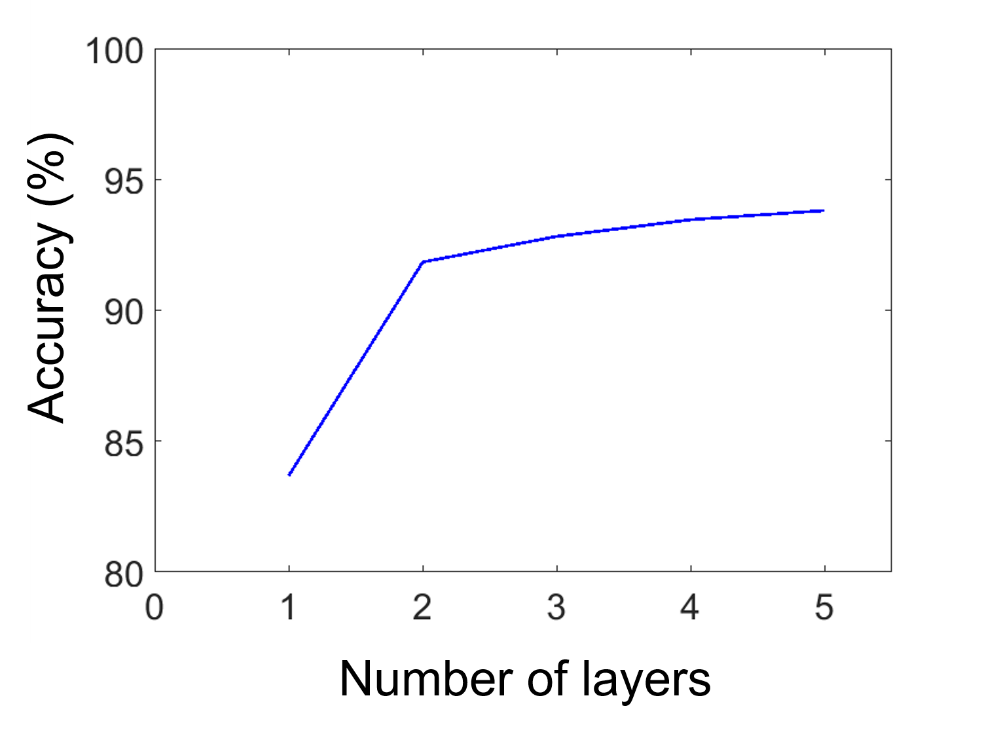


**Figure S3**. Performance of multilayer MLDs for the classification of nine classes of handwritten letters as a function of the number of layers separated in space. The systems were trained for 10 epochs with a training dataset of 54000 images. The testing dataset comprises 9000. The operative wavelength λ was 785 nm, the pixel diameter was 419 nm, the distances from input and output plane and between the layers was 31.4 μm and the absorption coefficient α was 0.02.


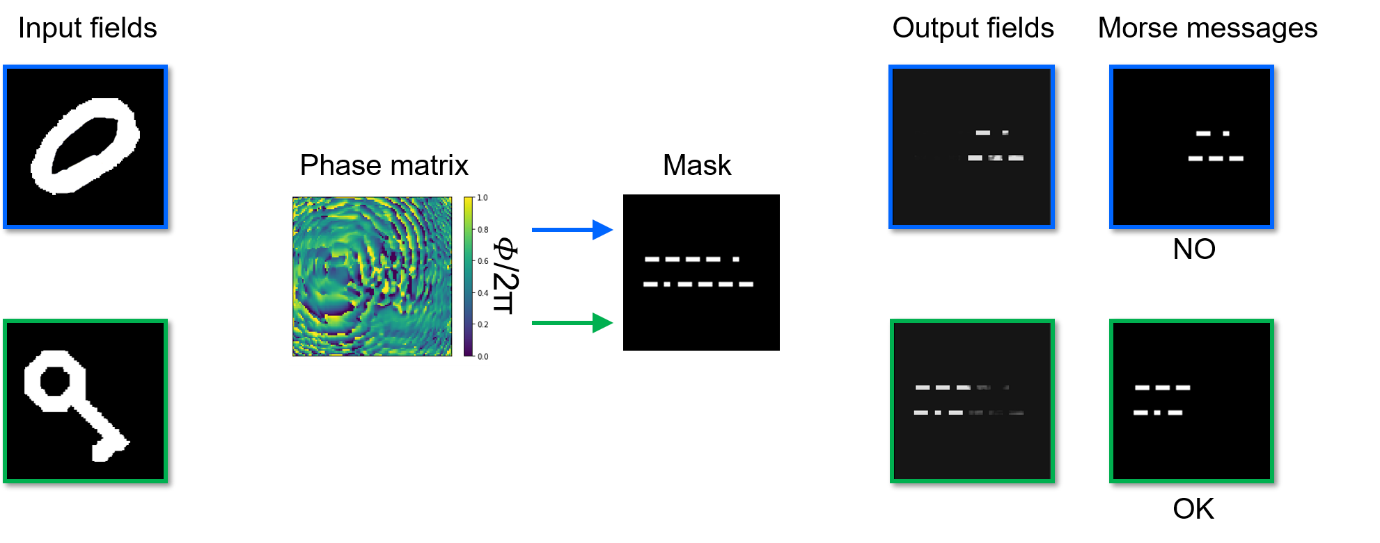


**Figure S4**. Numerical simulation showing an MLD performing symmetric decryption. The MLD considered here can recognize the correct key image against 6000 other objects belonging to 3 different classes of handwritten letters. The MLD maps the key and the letters into “OK” and “NO” messages expressed in Morse alphabet, respectively. In this case, the encrypted message is unrecognizable from the mask.


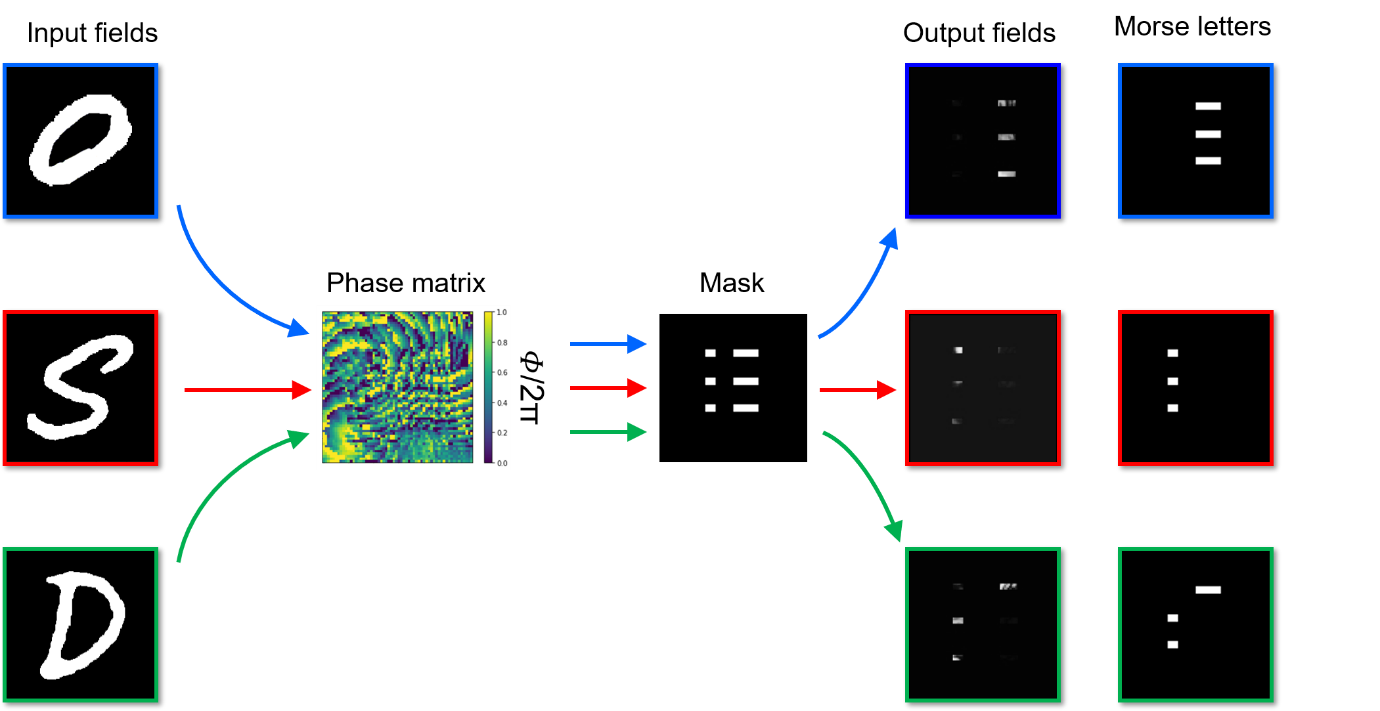


**Figure S5**. Numerical simulation showing an MLD performing asymmetric decryption. Images of different handwritten letters belonging to three different letter classes O, S, and D are encoded into amplitude fields. If images are propagated through the phase matrix, they are diffracted into three output images representing the corresponding class of letters in Morse alphabet. In practice, a private key would be used to retrieve the Morse information.


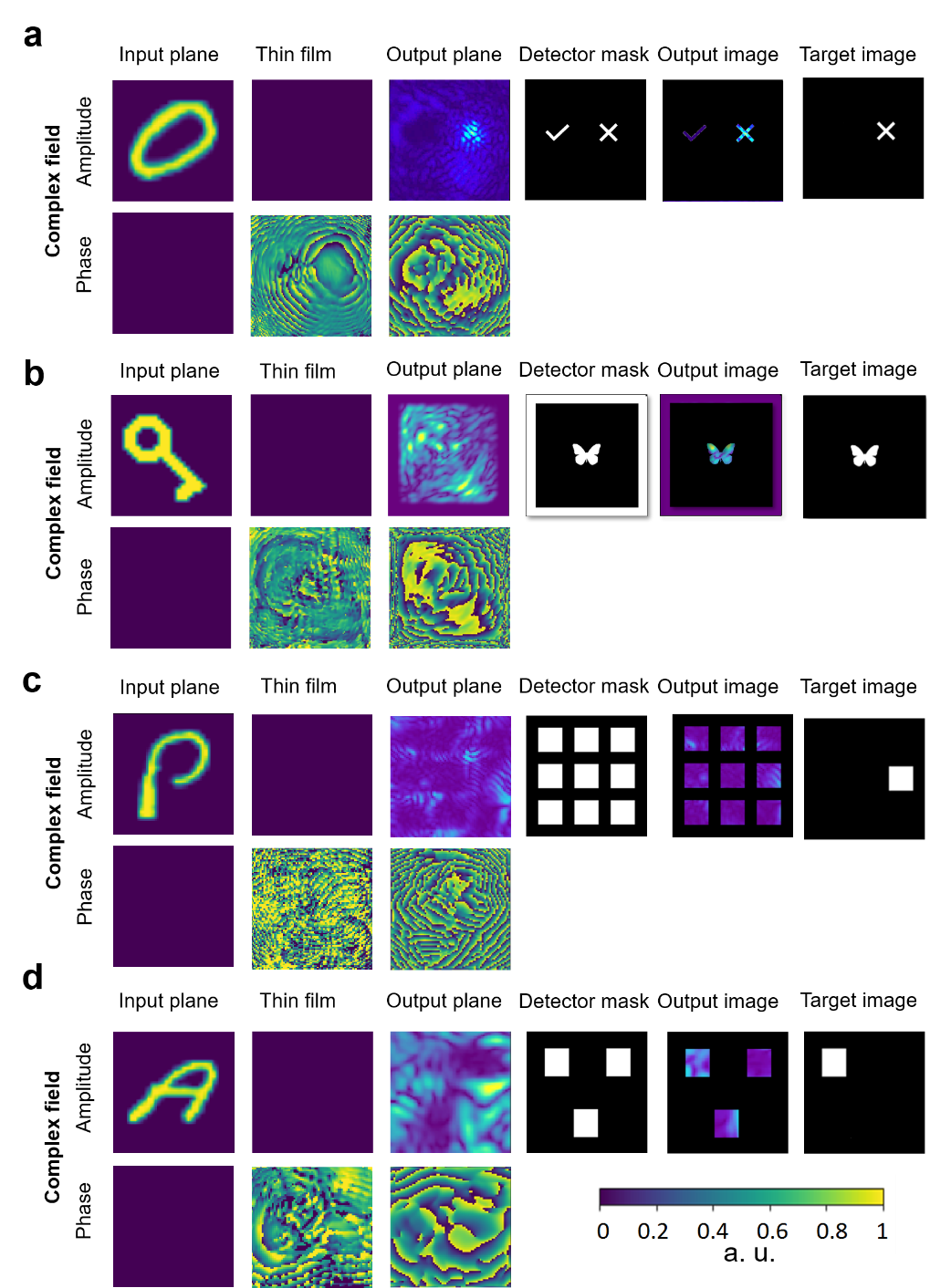


**Figure S6**. The input information was encoded in the amplitude channel of the input plane, for which, we consider coherent illumination. Complex fields in input and output, phase matrixes, detector masks and target images are shown for MLD-T (**a**), MLD-B (**b**), 9-MLD (**c**) and 3-MLD (**d**). The 9-MLD in this case consisted of two compact layers (phase modulation of each layer 0 - 0.6∙2π) of 6400 pixels each, with a diameter of 419 µm, D1 = 70.7 μm and D2 = 31.4 μm. The 3-MLD consisted of a single layer (phase modulation 0-2π) of 4900 pixels with a diameter of 413 µm, D1 = D2 = 55.0 μm. Both MLD-T and MLD-B consisted of a single layer (phase modulation 0-2π) of 10000 pixels with a diameter of 413 µm, D1 = D2 = 47.1 μm. The operative wavelength λ was 785 nm and the absorption coefficient α was 0.02. The systems were trained for 10 epochs with a training dataset of 18000 images in the case of 3-MLD, 54000 images in the case of 9-MLD, and 24000 images in the case of MLD-T and MLD-B.


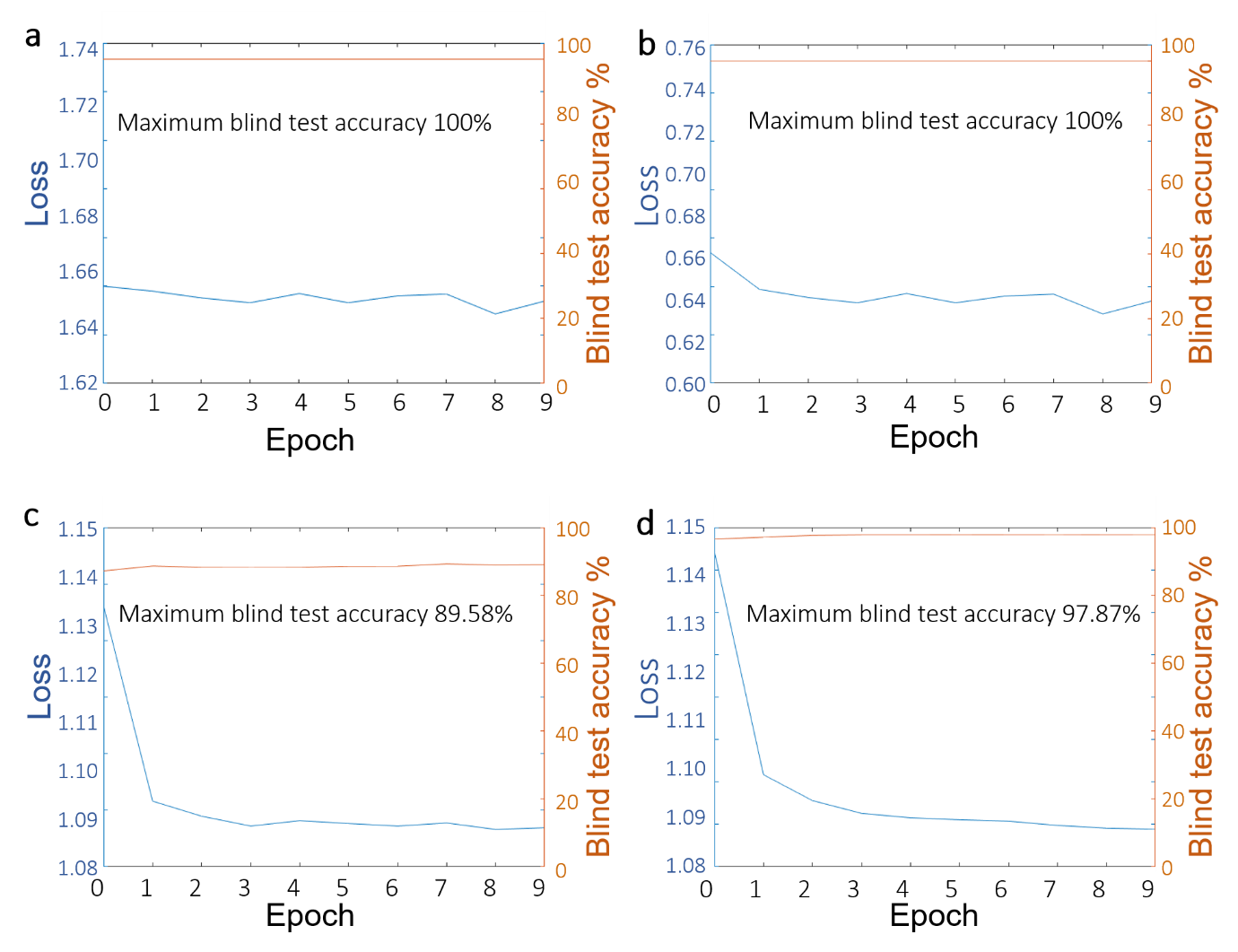


**Figure S7**. Training convergence plot for MLD-T (**a**), MLD-B (**b**), 9-MLD (**c**) and 3-MLD (**d**), reported in **Figure S6**. The plot shows the loss values (blue) and the letters classification accuracy for the testing dataset (1000 images per letter or image category) as a function of the epoch number. From the plots it emerges that symmetric encryption algorithms are computationally more efficient in comparison to asymmetric algorithms.


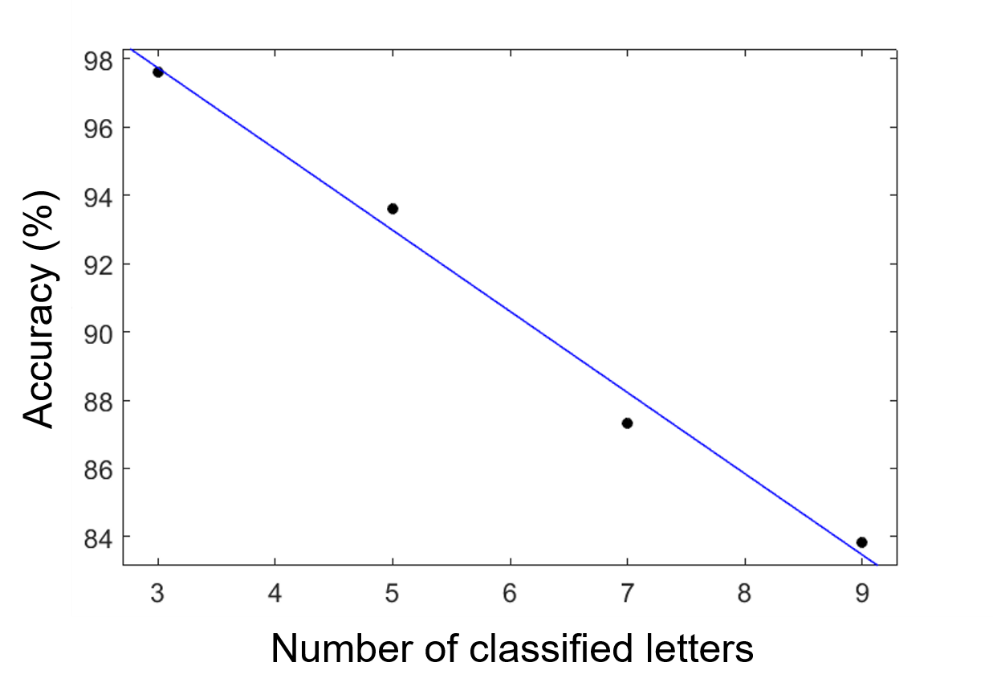


**Figure S8.** Blind test classification accuracy after 10 epochs as a function of the number of classes of letters to encrypt. The blue line is the linear fitting (Matlab R2018b) of the results of numerical simulations (black circles). The operative wavelength λ was 785 nm, the pixel diameter was 393 nm, the distances from input and output plane D1 = D2 = 31.4 μm and the absorption coefficient α was 0.02.


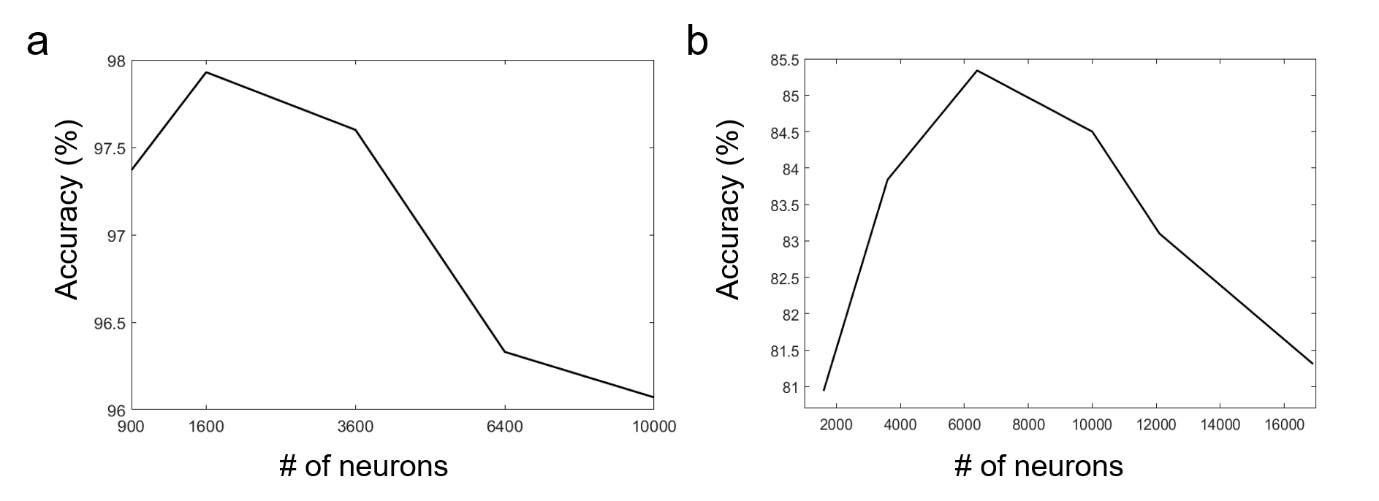


**Figure S9**. Performance of MLDs for the decryption of three (3-MLD) (**a**) and nine (9-MLD) (**b**) classes of handwritten letters as a function of the number of pixels. The MLDs were trained for 10 epochs with a training dataset of 18000 and 54000 images for 3-MLD and 9-MLD, respectively. The testing datasets comprise 3000 and 9000 images for 3-MLD and 9-MLD, respectively. The operative wavelength λ was 785 nm, the pixel diameter was 393 nm, the distances from input and output plane D1 = D2 = 31.4 μm and the absorption coefficient α was 0.02.


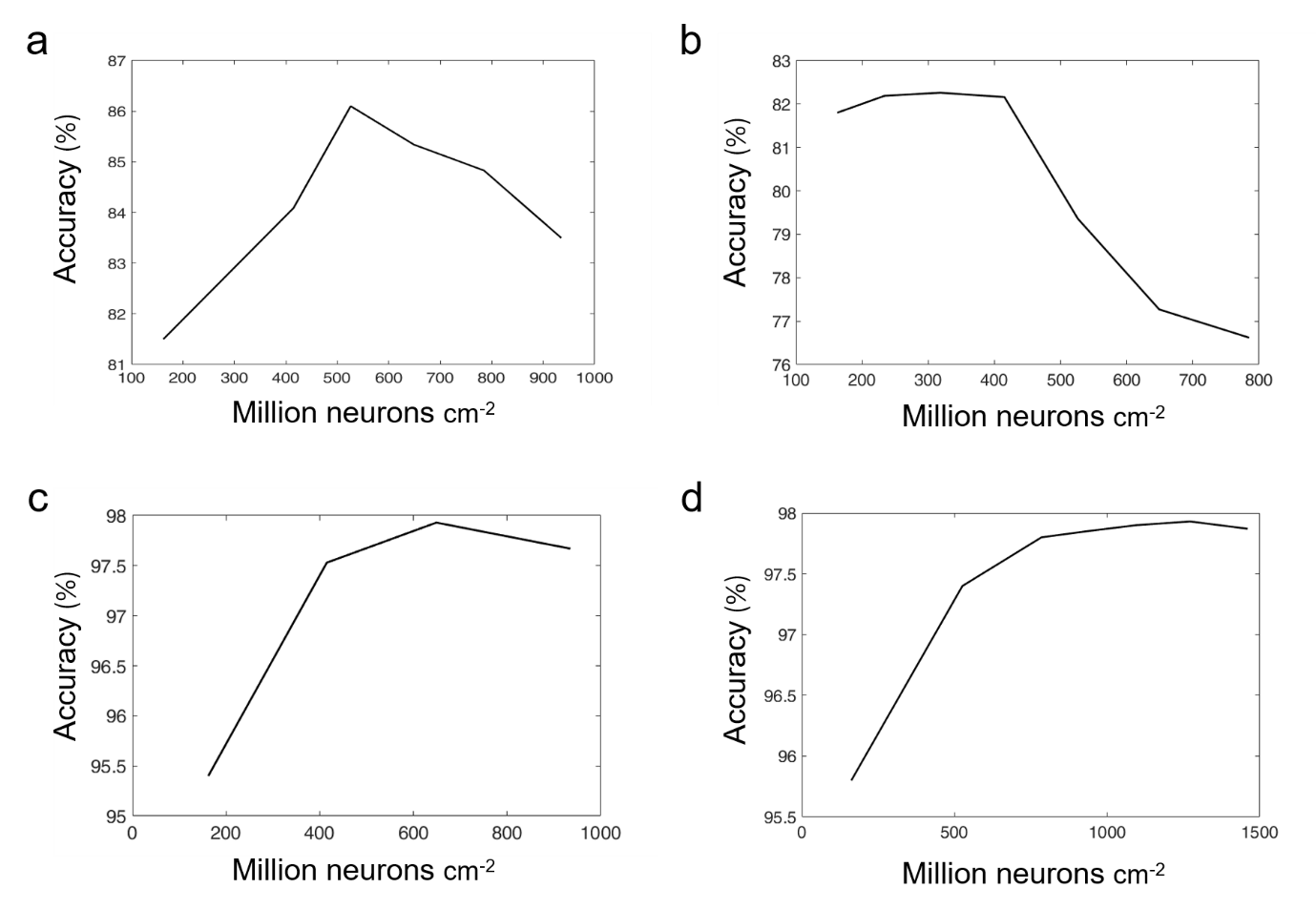


**Figure S10**. Performance of 9-MLD with 6400 (**a**) and 900 (**b**) pixels as a function of the neuron density. Performance of 3-MLD with 1600 (**c**) and 3600 (**d**) pixels as a function of the pixel dimension. The MLDs were trained for 10 epochs with a training dataset of 18000 and 54000 images for 3-MLD and 9-MLD, respectively. The testing datasets comprise 3000 and 9000 images for 3-MLD and 9-MLD, respectively. The operative wavelength λ was 785 nm, the distances from input and output plane D1 = D2 = 31.4 μm and the absorption coefficient α was 0.02.


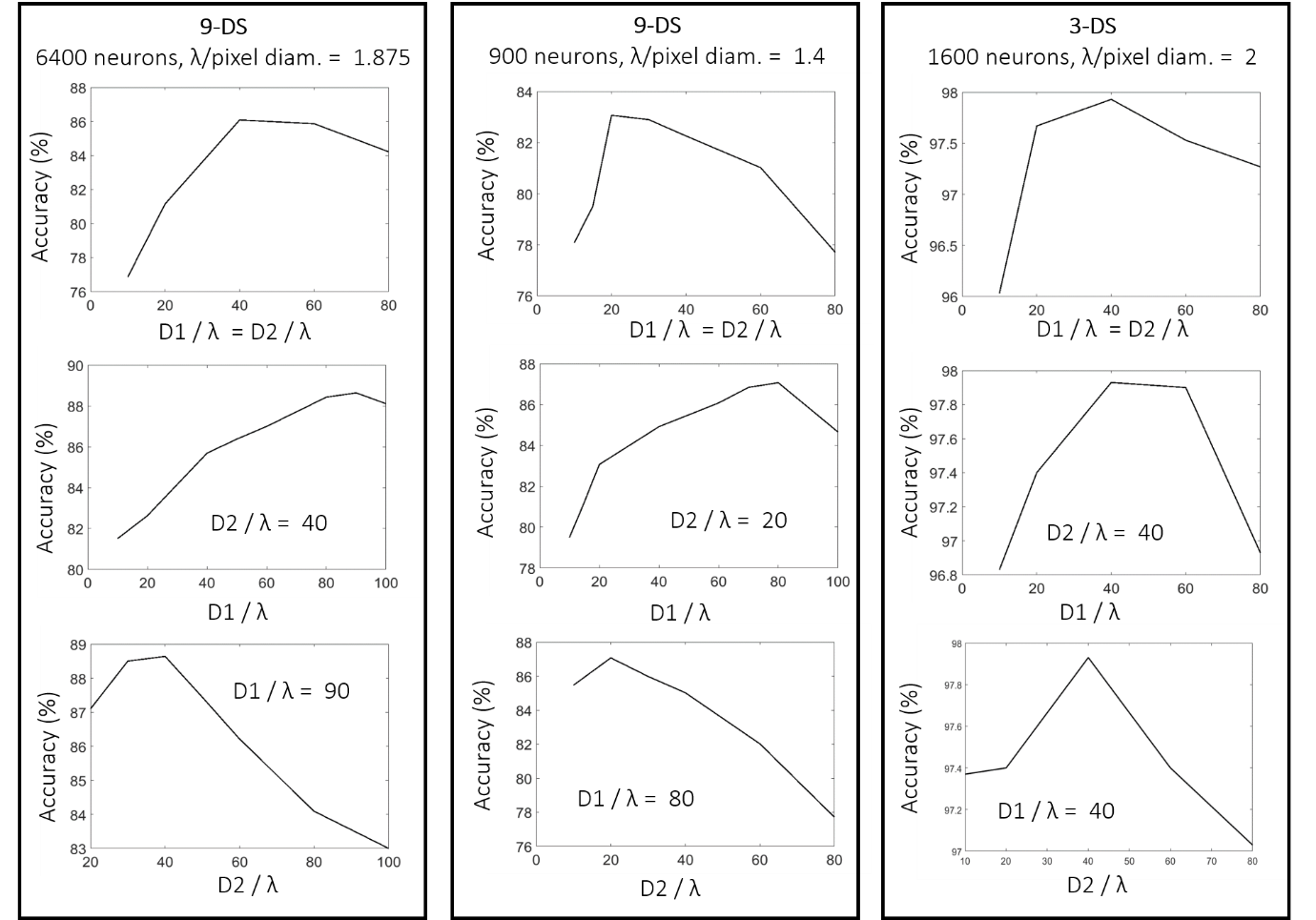


**Figure S11**. Performance of 9-MLD and 3-MLD with different number of pixels and pixel size as a function of the distances from input and output plane, D1 and D2 respectively. The MLDs were trained for 10 epochs with a training dataset of 18000 and 54000 images for 3-MLD and 9-MLD, respectively. The testing datasets comprise 3000 and 9000 images for 3-MLD and 9-MLD, respectively. The operative wavelength λ was 785 nm and the absorption coefficient α was 0.02.


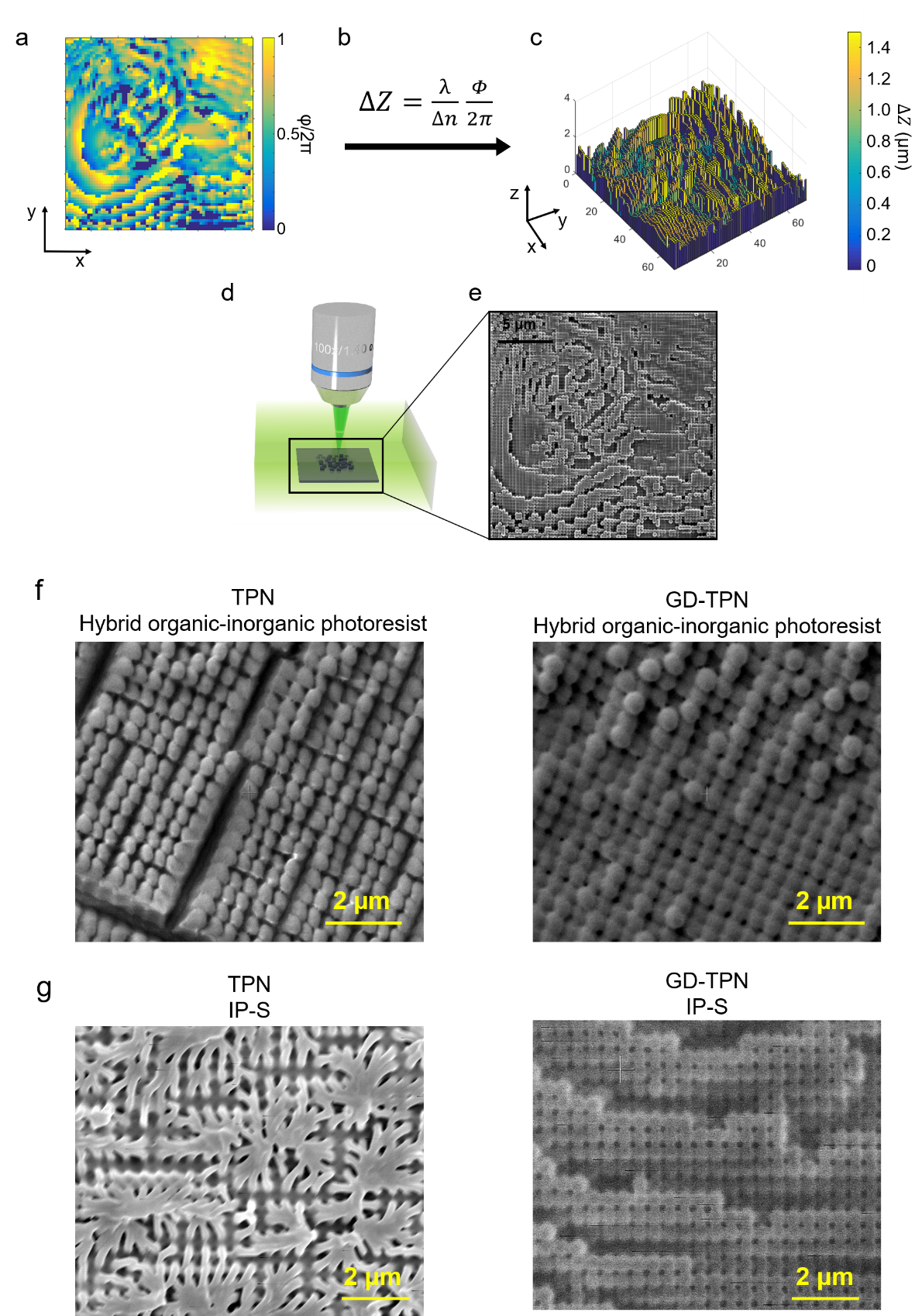


**Figure S12**. At the end of the computer-based learning process, phase matrixes (**a**) were converted, using equation **b**, into a relative height map (**c**). Δ𝑛 is the refractive index difference between the 3D printing material (**Figure S12**) and air, and λ is the operative wavelength. Using GD-TPN method (**d**) we were able to 3D-print the MLD designs (**e**). SEM images of 3D nano-printed phase masks printed via TPN and GD-TPN using a custom-made photoresist^3^ (**f**) and commercial IP-S photoresist (**g**).


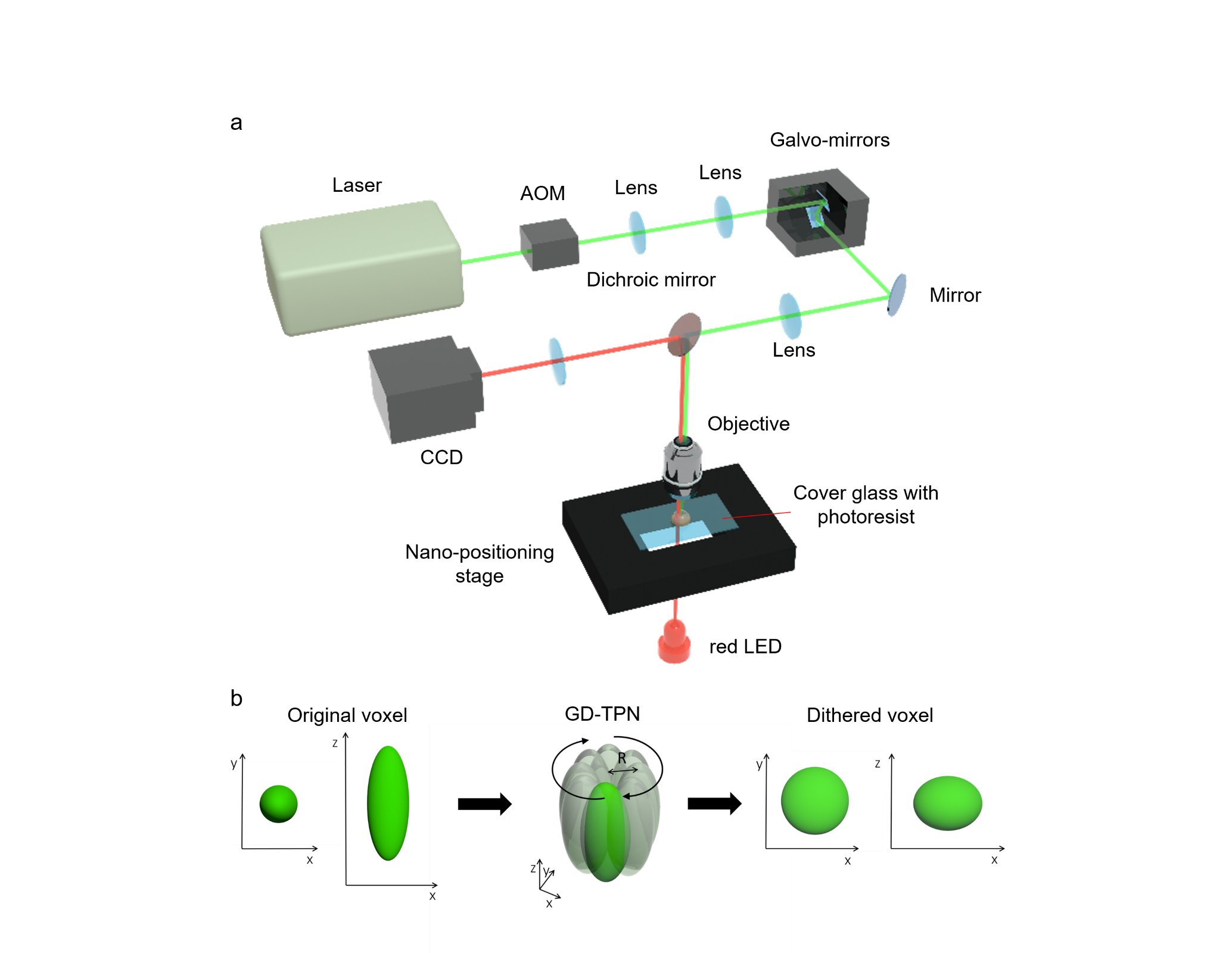


**Figure S13**. **a**) Schematic of the GD-TPL setup described in the Supplementary Methods. **b**) Illustration of circular dithered correction applied by galvo-mirrors. The galvo-mirrors force a circular motion of the laser beam in the xy plane. This causes the fabrication voxel to become shorter in the Z direction, improving the stability of the fabrication voxel, the overall resolution of the 3D fabrication method and leading to correction of voxel asymmetry.


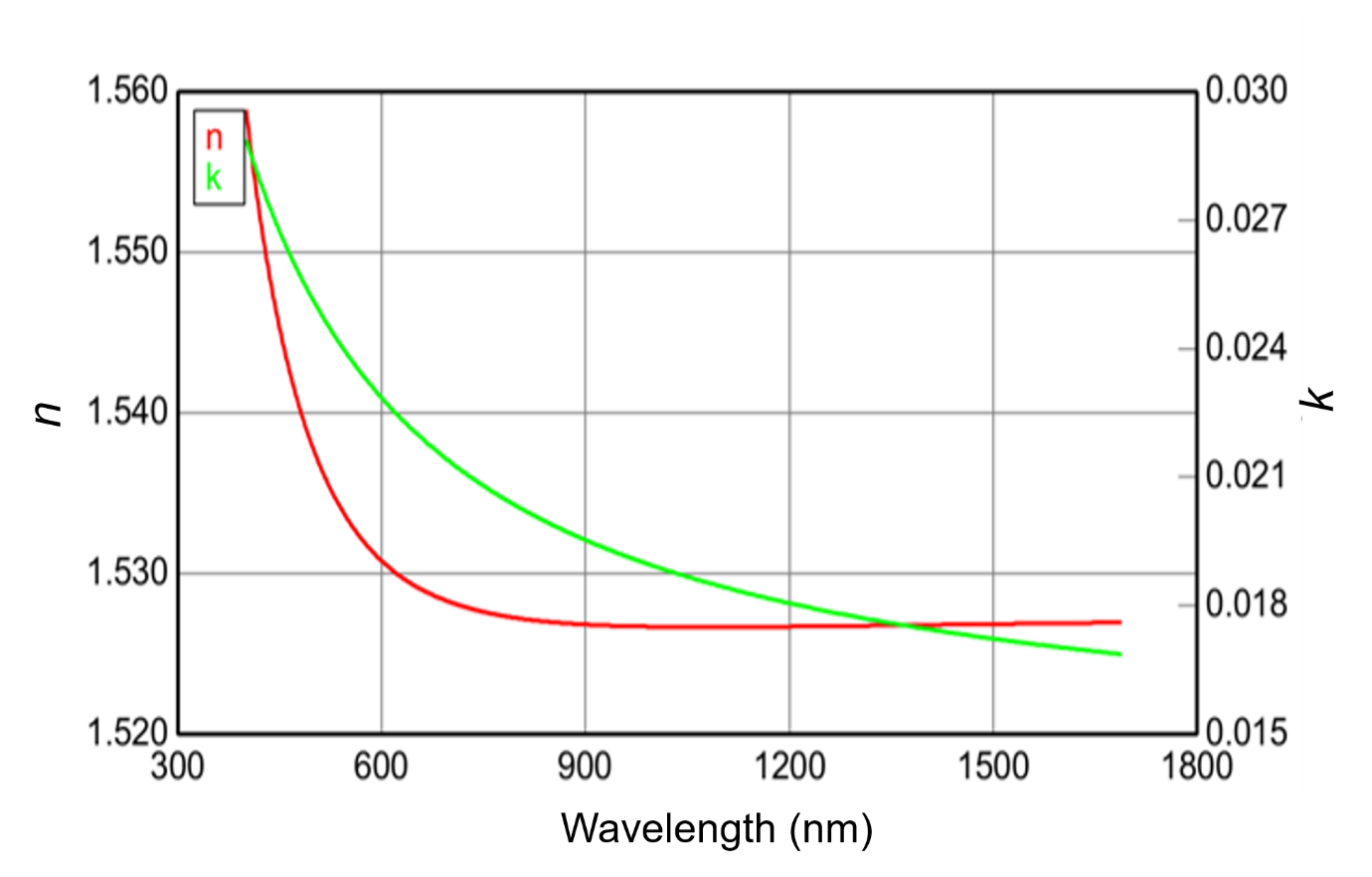


**Figure S14**. Refractive index (*n*) and extinction coefficient (*k*) of the custom-made zirconium-based photoresist used to print the MLDs design using the GD-TPN.


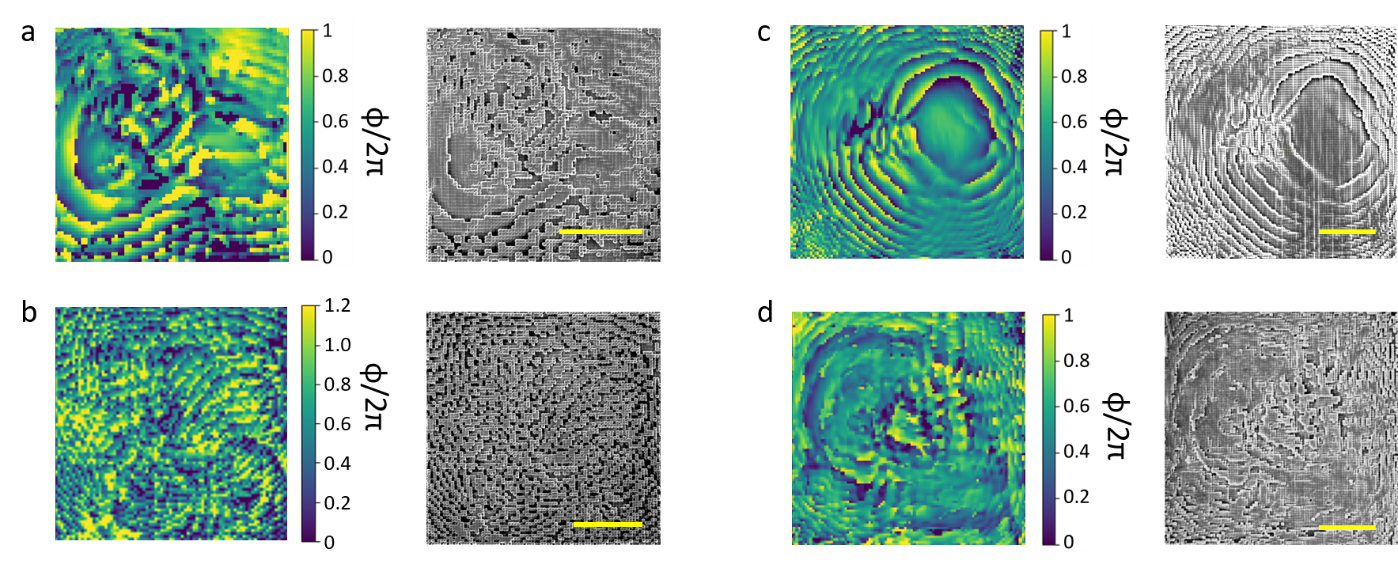


**Figure S15**. Optimised phase matrixes and scanning electron microscopy images of the corresponding 3D-printed 3-MLD (**a**), 9-MLD (**b**), MLD-T (**c**) and MLD-B (**d**) presented in **Figure S11**. The scale bars are 20 µm.


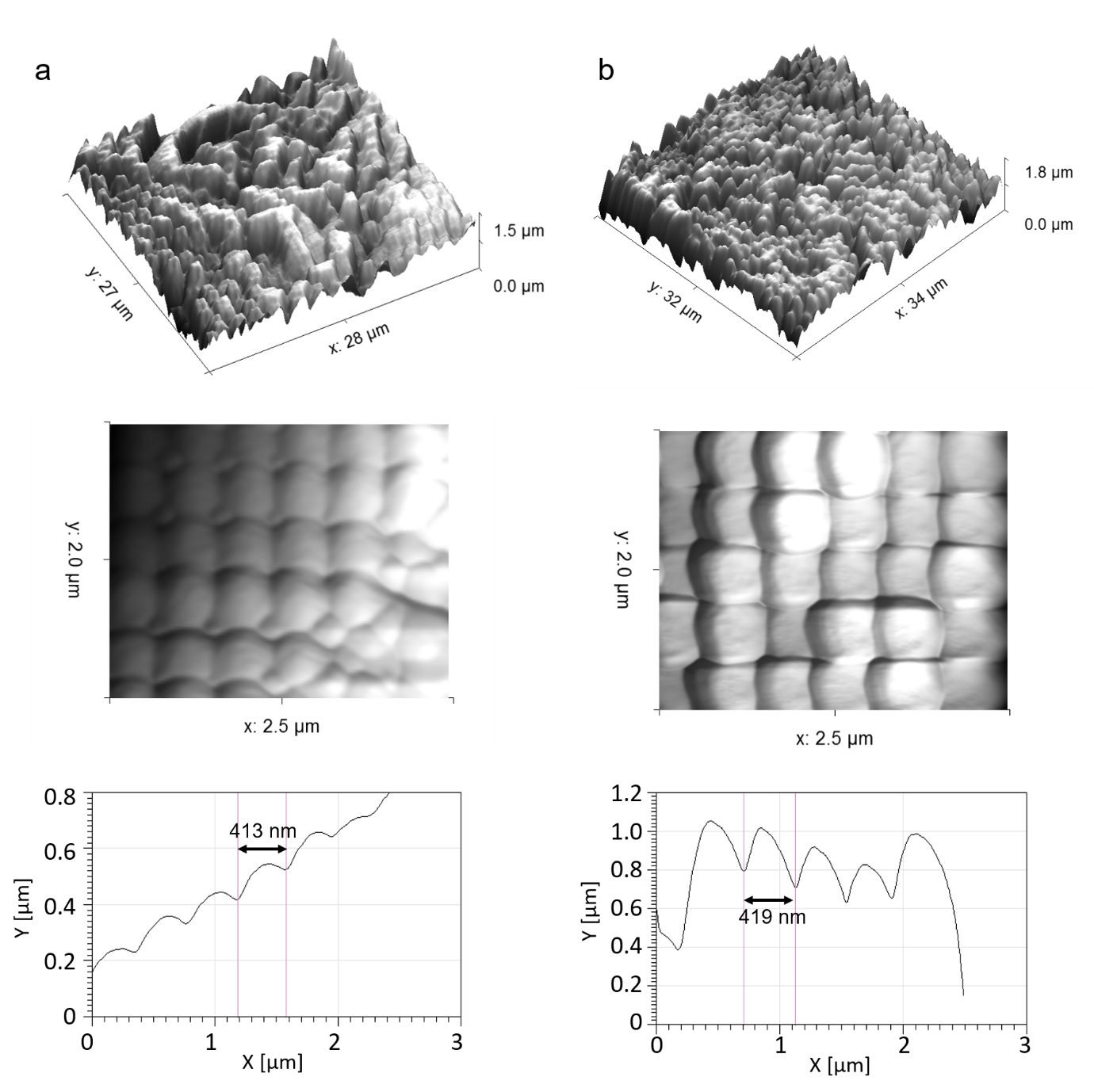


**Figure S16**. AFM 3D profile, topographical image and line profile of a section of the 3D-printed 9-MLD shown in **Figure S16**.


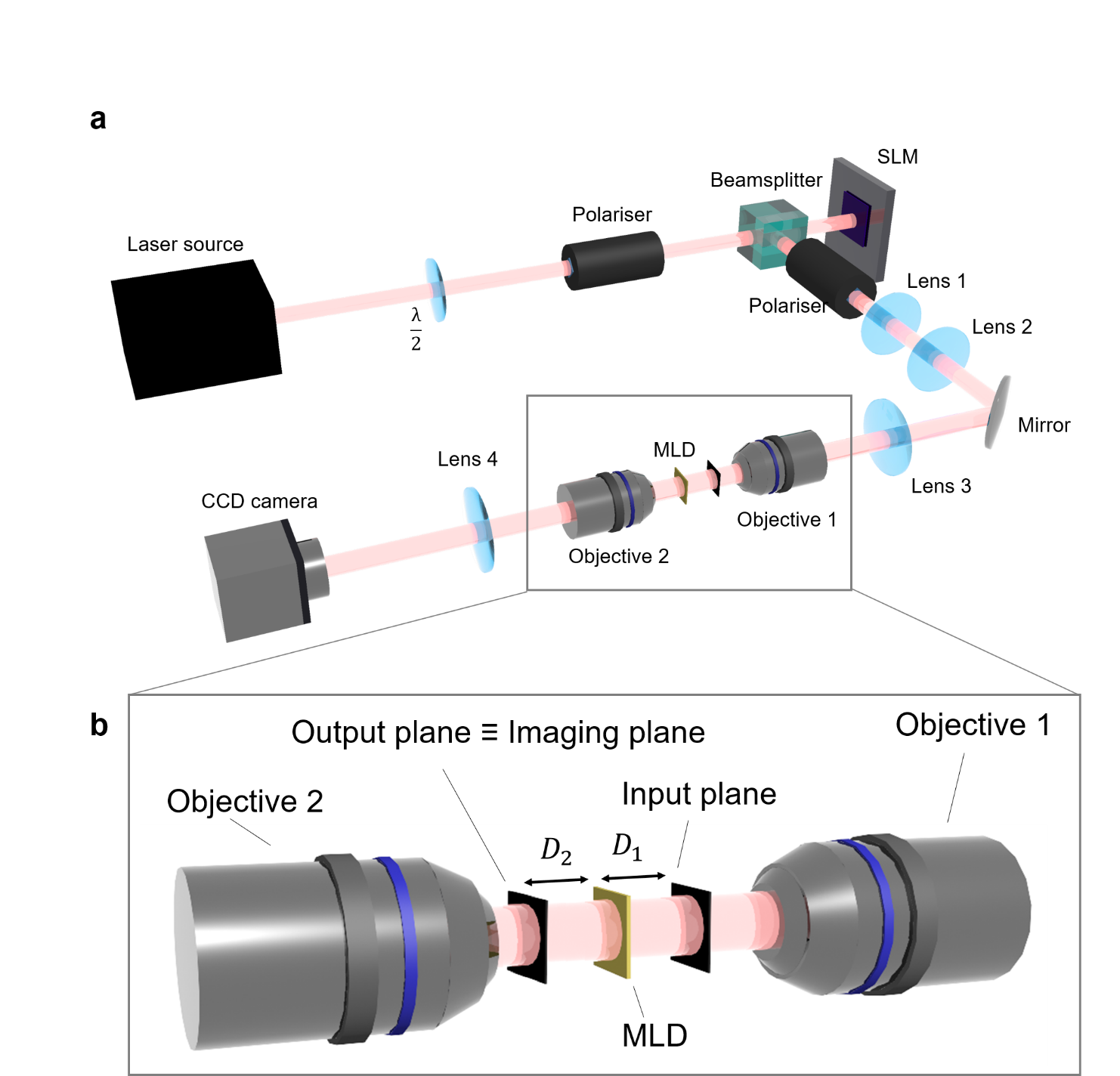


**Figure S17**. **a**) Schematic of the characterization setup. We used a Thorlabs OBIS 785 nm laser source to generate a continuous wave radiation to test the MLDs performance. The input handwritten letter was generated using a spatial light modulator (SLM) (LCOS-SLM X13138-07, Hamamatsu) and imaged at the input plane of the MLD using two 4f systems. The output image of the MLD was detected using a 4f system and an Basler ace acA2040-90uc CCD camera. The objectives are mounted on two linear translation stages (two Thorlabs standard micrometres PT1 to achieve precise alignment in the xy plane) and the sample holder is mounted on a 3D linear translation stage (Thorlabs PT3/M - 25.0 mm XYZ Translation Stage) to facilitate the alignment and minimise the incident angle offset. **b**) Schematic detail of the experimental characterization setup (**Figure S18**). Two objectives (Olympus UPLANFL N, 60X 0.9NA) are used to focus the handwritten letter at the input plane and to image the output plane of the MLDs.


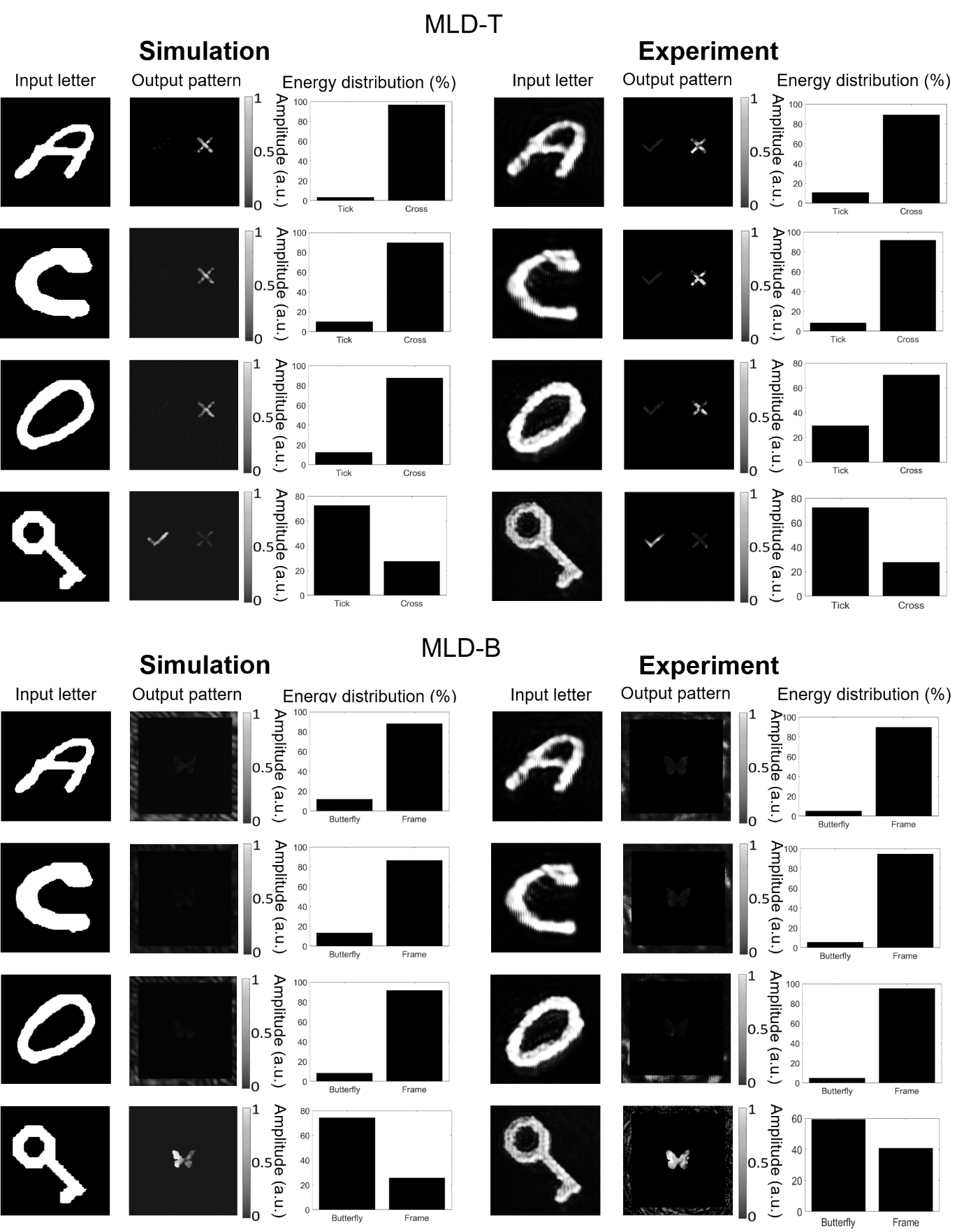


**Figure S18**. Simulated (left) and experimental (right) input field and corresponding output pattern and energy distribution percentage relative to three different handwritten letters and a key image successfully decrypted with our 3D-printed MLD-T (top) and MLD-B (bottom) from **Figure S7a** and **S7b**.

**
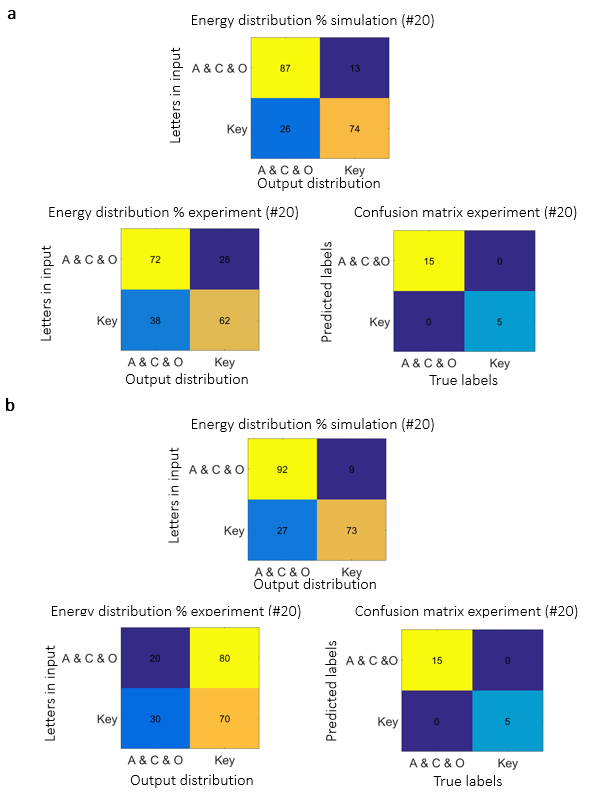
**

**Figure S19**. Analysis of MLD-T from **Figure S7a**. **a**) Theoretical energy distribution percentage table, experimental energy distribution percentage table and experimental confusion matrix obtained using 20 different handwritten letters (5 for each letter and image class) selected among the successfully classified testing images. Analysis of MLD-B from **Figure S7b**. **b**) Theoretical energy distribution percentage table, experimental energy distribution percentage table and experimental confusion matrix obtained using 20 different handwritten letters (5 for each letter and image class) selected among the successfully classified testing images.


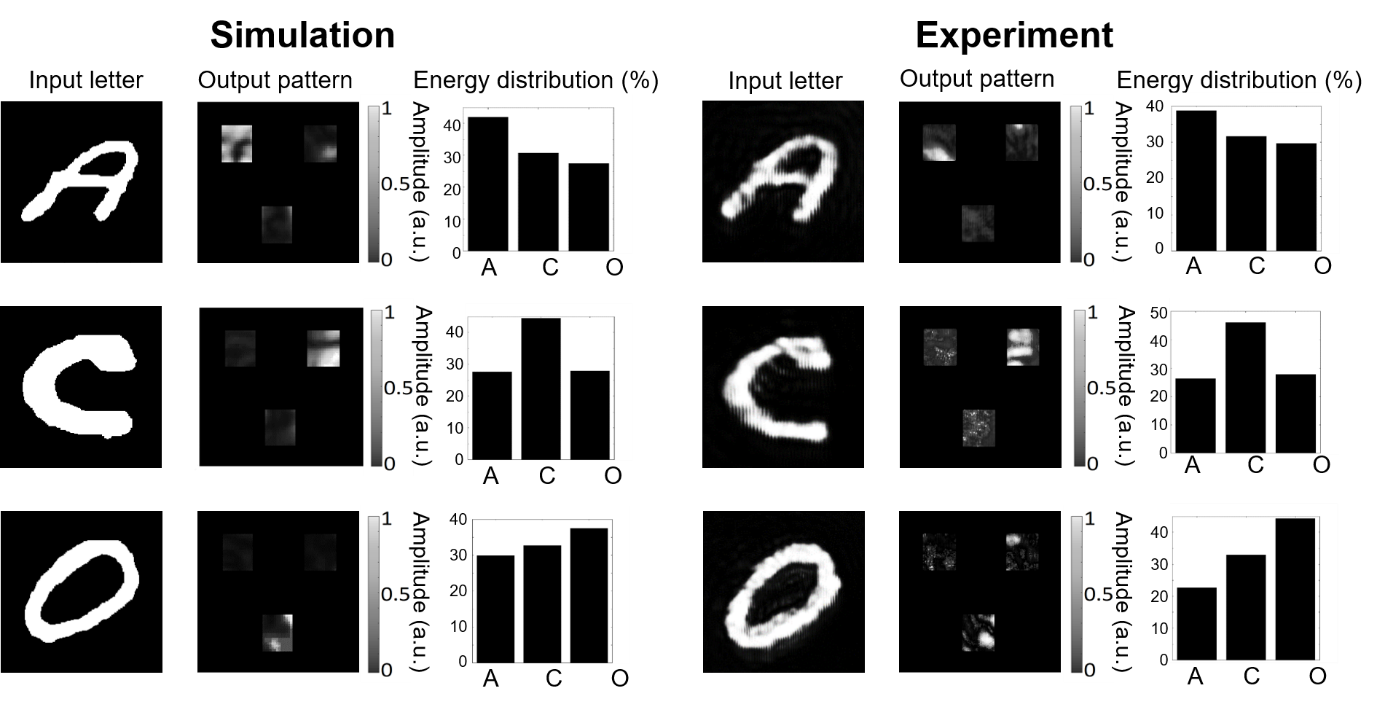


**Figure S20**. Examples of the theoretical (left) and the experimental (right) decryption results achieved with our 3D-printed 3-MLD from **Figure S7d**. The energy distribution percentage corresponding to each digit at the output plane shows that 3-MLD has the maximum energy focused on the target detector region of each letter.


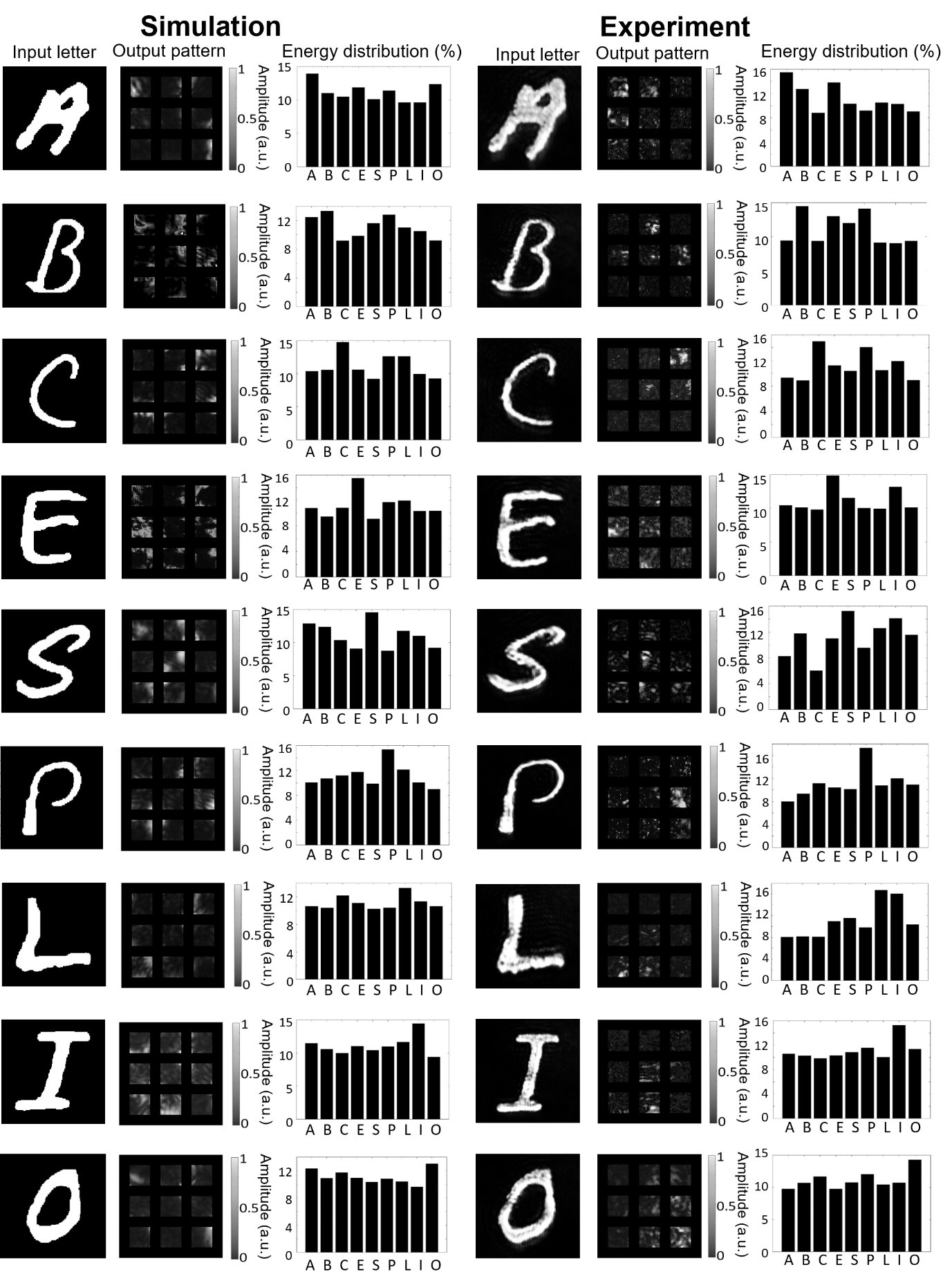


**Figure S21**. Simulated (left) and experimental (right) input field and corresponding output pattern and energy distribution percentage relative to 9 different handwritten letters successfully decrypted with our 3D-printed 9-MLD from **Figure S7c**.


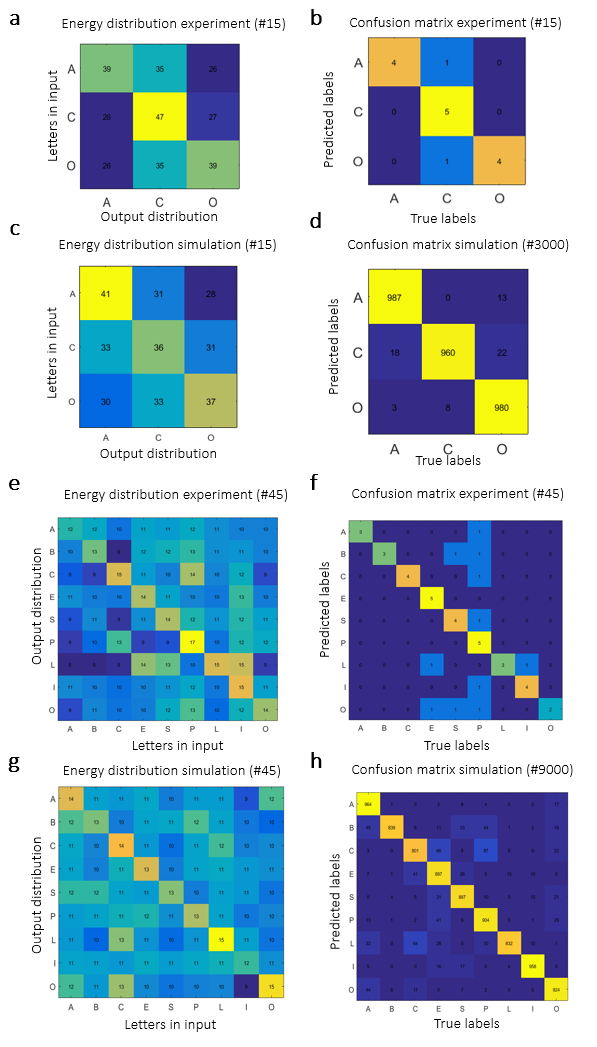


**Figure S22**. Analysis of 3-MLD from **Figure S7d**. Experimental energy distribution percentage table (**a**) and experimental confusion matrix (**b**) obtained using 15 different handwritten letters (5 for each letter class) selected among the successfully classified testing images. Theoretical energy distribution percentage table (**c**) and theoretical confusion matrix (**d**) obtained using the same 15 different handwritten letters used in (**a**) and 3000 different handwritten letters (1000 for each letter class), respectively. Analysis of 9-MLD from **Figure S7c**. Experimental energy distribution percentage table (**e**) and experimental confusion matrix (**f**) obtained using 45 different handwritten letters (5 for each letter class) selected among the successfully classified testing images. Theoretical energy distribution percentage table (**g**) and theoretical confusion matrix (**h**) obtained using the same 45 different handwritten letters used in (**e**) and 9000 different handwritten letters (1000 for each letter class), respectively.


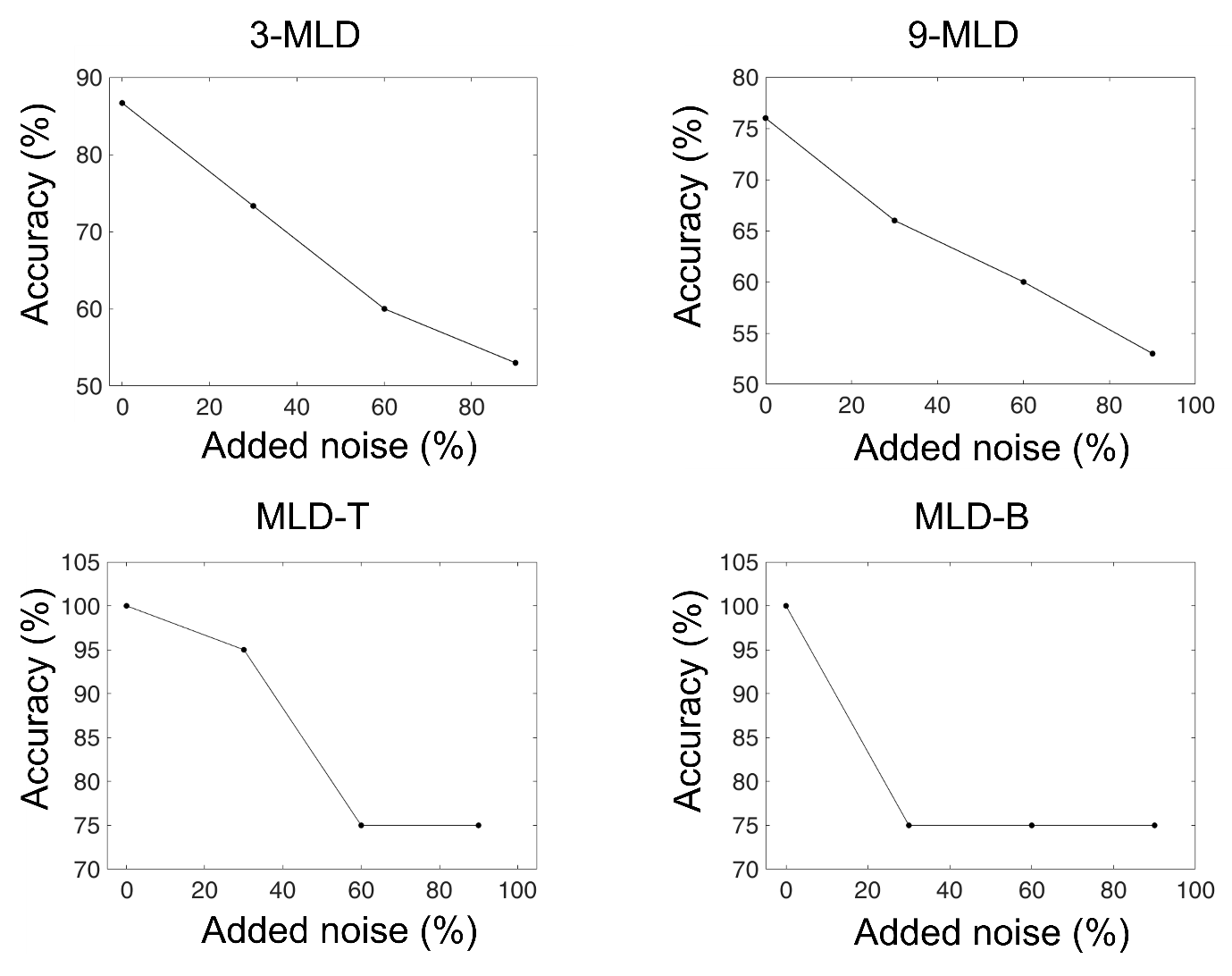


**Figure S23**. Study of the role of noise in our experimental characterization results. The plots show the accuracy of our MLDs as a function of the added normalised noise in percentage. The results are obtained adding normalised random noise to the images used for the experimental characterization.


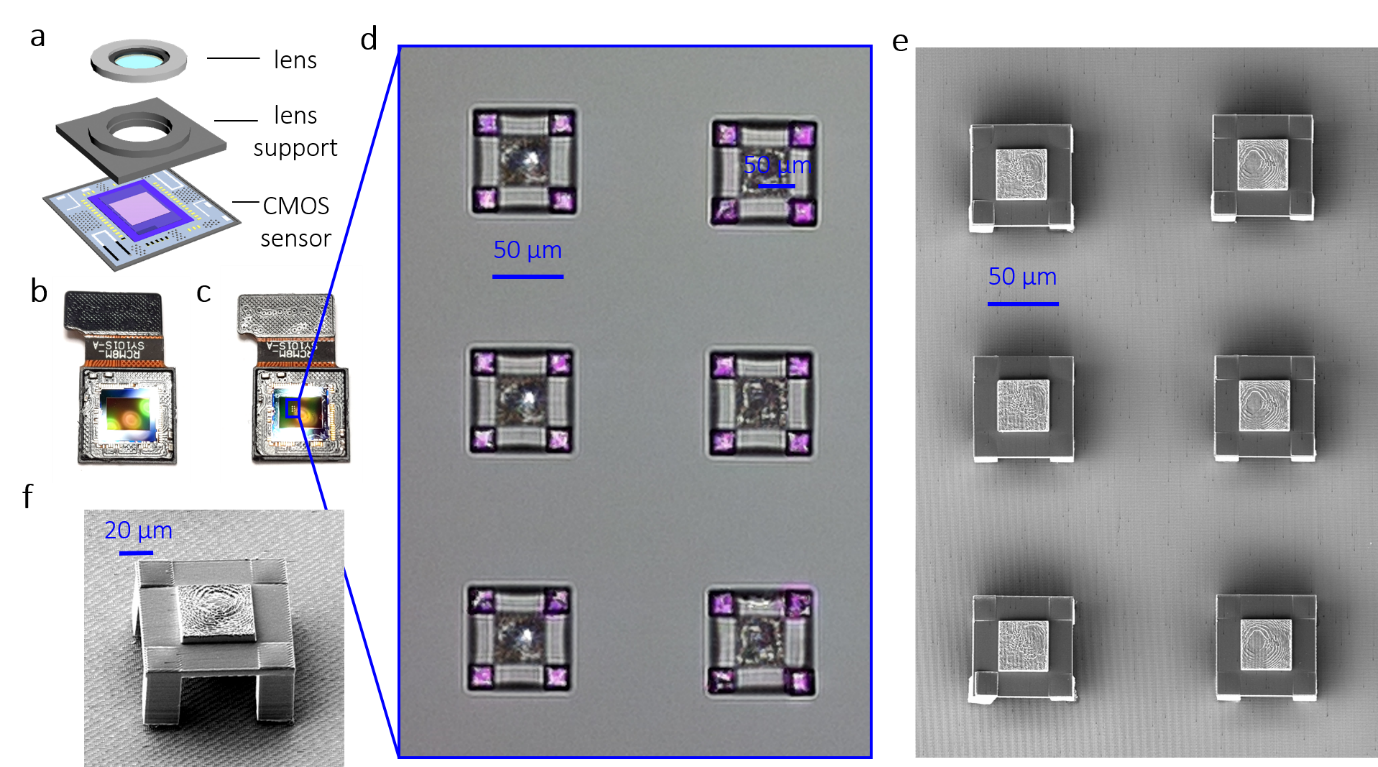


**Figure S24**. **a**) Diagram of the components of a Sony IMX219 NoIR CMOS image sensor from a Raspberry Pi Camera Module. From the top, lens, lens support, CMOS sensor. **b**) Photograph of the CMOS sensor before TPN. **c**) Photograph of the CMOS sensor with an array of 2 × 3 MLDs printed directly on the sensor via TPN. The different colour of the connection unit (top part of the CMOS detector) is due to a different reflection of the light due to different inclination. **d**) Scanning electron microscopy (SEM) image of the MLDs array, top view. **e**) Image collected with the CMOS sensor of the MLDs array. The scale bar is 44 pixels (50 µm). Images **d** and **e** show the same sample, however, image e is the mirror image of image **d** since the SEM image shows the top view, while image **e** shows is taken with the CMOS sensor underneath the structures and shows the view from the bottom. **f**) SEM image of an MLD printed on CMOS camera, side view. The diffractive layer is printed on a table-like support and the height of the pillars is 47.1 μm. The image is take after characterising the structure, proving the stability of the design and the potential of the MLD to be used for several measurements.


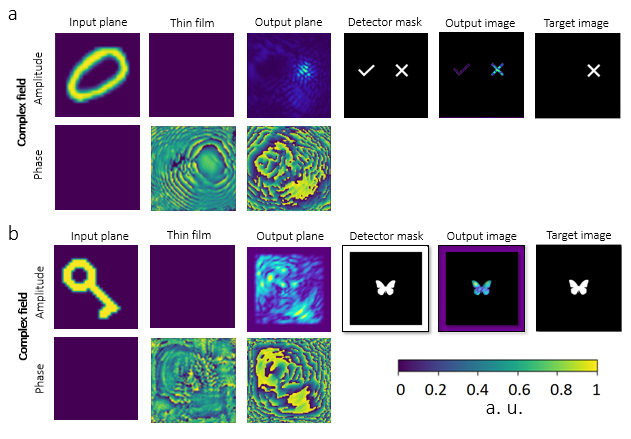


**Figure S25**. Complex fields in input and output, phase matrixes, detector masks and target images are shown for MLD-T_IPS_ (**a**) and MLD-B_IPS_ (**b**). Both the MLDs consisted of a single layer (phase modulation 0-2π) of 10000 pixels with a diameter of 413 nm, D1 = D2 = 47.1 μm. The operative wavelength λ was 785 nm and the absorption coefficient α was approximated to 0. The systems were trained for 10 epochs with a training dataset of 24000 images.


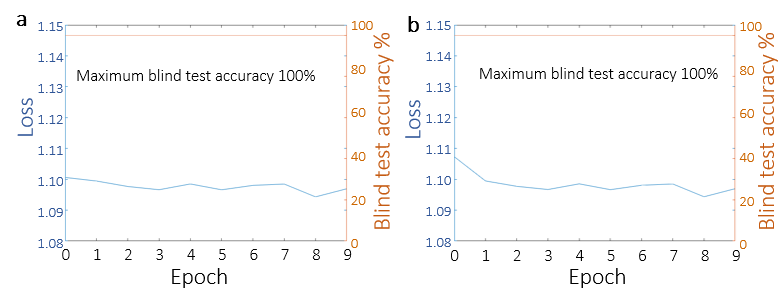


**Figure S26**. Training convergence plot for MLD-T_IPS_ (**a**) ad MLD-B_IPS_ (**b**) reported in **Figure S24**. The plot shows the loss values (blue) and the letters classification accuracy for the testing dataset (1000 images per letter or image category) as a function of the epoch number.


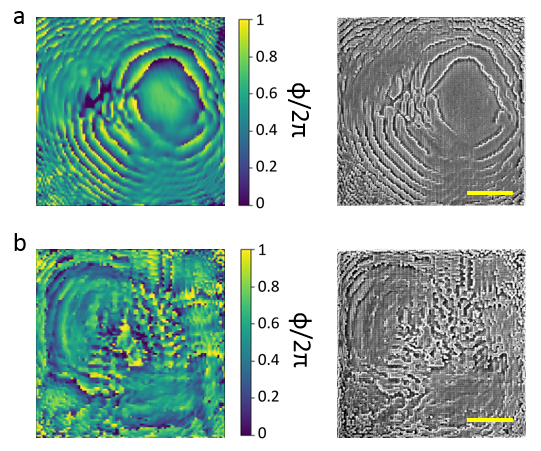


**Figure S27**. Optimised phase matrixes and scanning electron microscopy images of the corresponding 3D-printed MLD-T_IPS_ (**a**) and MLD-B_IPS_ (**b**) presented in **Figure S25**. The scale bars are 20 µm.


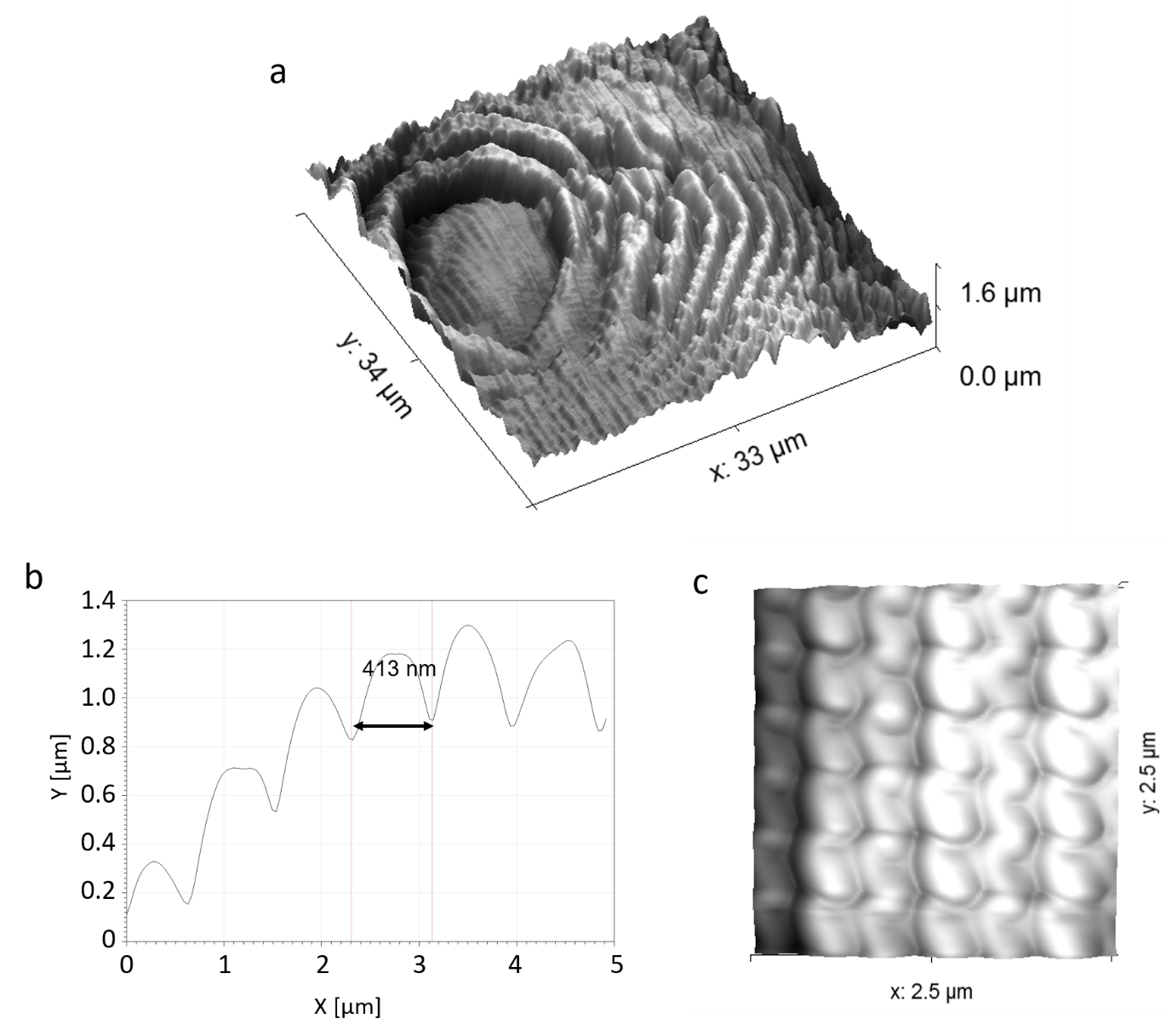


**Figure S28**. AFM 3D profile, topographical image and line profile of a section of the 3D-printed MLD-T_IPS_ shown in **Figure S26a**.


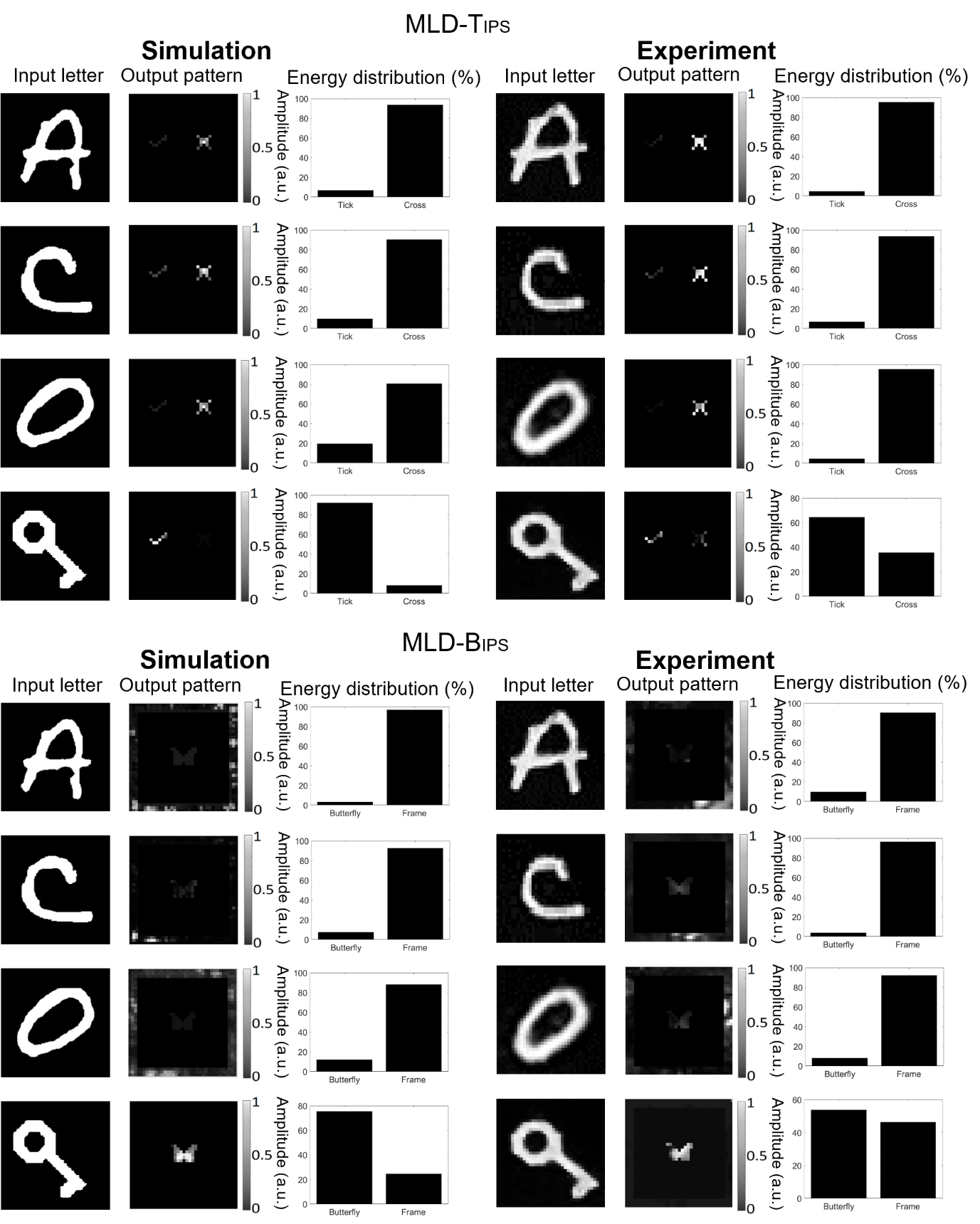


**Figure S29**. Theoretical (left) and experimental (right) input field and corresponding output pattern and energy distribution percentage relative to three different handwritten letters and the decryption key, successfully decrypted with our 3D-printed MLD-T_IPS_ (**top**) and MLD-B_IPS_ (**bottom**).


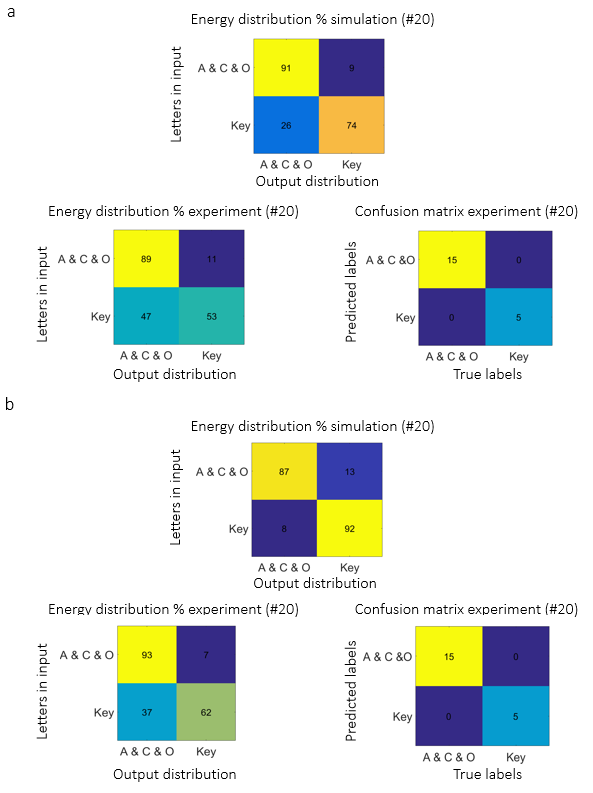


**Figure S30**. Analysis of MLD-T_IPS_ design. **a**) Theoretical energy distribution percentage table, experimental energy distribution percentage table and experimental confusion matrix obtained using 20 different handwritten letters (5 for each letter and image class) selected among the successfully classified testing images. Analysis of MLD-B_IPS_ design. **b**) Theoretical energy distribution percentage table, experimental energy distribution percentage table and experimental confusion matrix obtained using 20 different handwritten letters (5 for each letter and image class) selected among the successfully classified testing images.

**Tables**


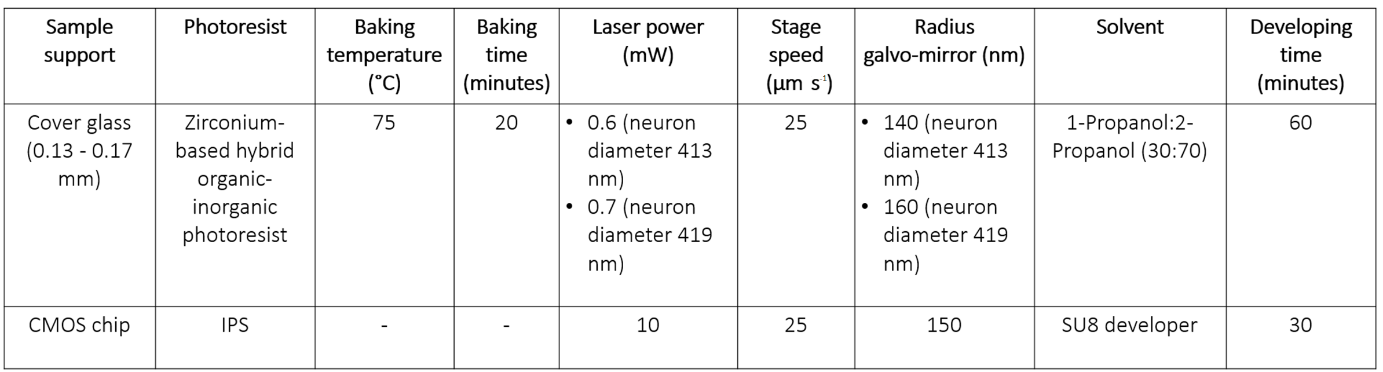


**Table ST1**. Details of the fabrication parameters used for the nanoprinting of the MLDs on cover glass and on CMOS chip.

| **MLD** | **Diffraction efficiency = I_d_ / I_i_ (%)** |
| --- | --- |
| 9-MLD | 12 % |
| 3-MLD | 15 % |
| MLD-B_IPS_ | 17 % |
| MLD-T | 4 % |
| MLD-B | 14 % |
| MLD-T_IPS_ | 5 % |

**Table ST2**. Experimental diffraction efficiency values of the MLDs characterized in this work.

.


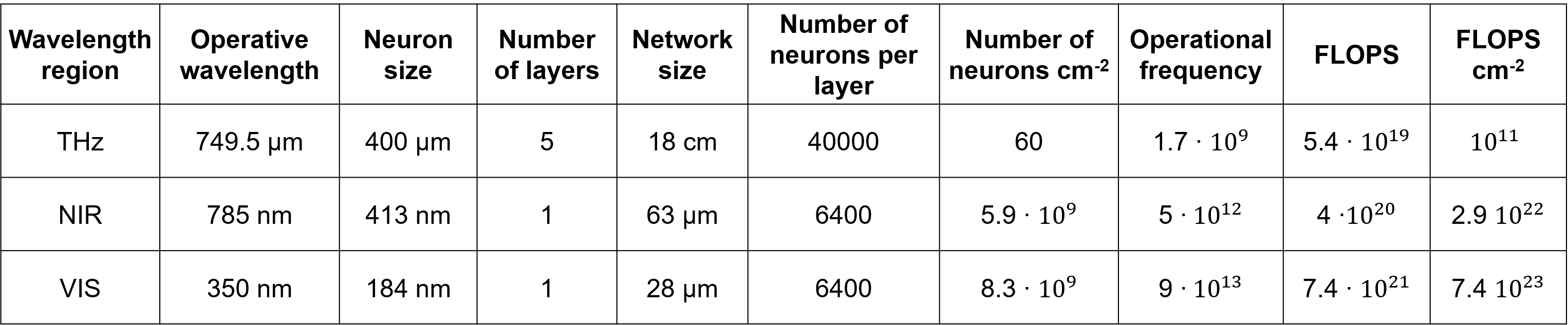


**Table ST3**. Operational and geometrical parameters, operational frequencies and FLOPS for diffractive neural networks operating in the THz, NIR and visible wavelength regions, calculated using Eq. 6 and Eq. 7.

**References**

1. Goodman, J. W. *Introduction to Fourier Optics. Third edition.* (Stanford University, 2005).

2. Lin, X. *et al.,* All-optical machine learning using diffractive deep neural networks. *Science* **361**, 1004–1008 (2018).

3. Terzaki, K. *et al.,* 3D conducting nanostructures fabricated using direct laser writing. *Optical Materials Express* **1**, 586–597 (2011).

4. Rosenblatt, F. *The Perceptron: A Perceiving and Recognizing Automaton*. Report 85-60-1, Cornell Aeronautical Laboratory, Buffalo, New York, (1957).

5. Kingma, D. P. & Ba, J., Adam: a method for stochastic optimization. arXiv:1412.6980 (2014).

6. Mengu, D., *et al.*, A. Analysis of diffractive optical neural networks and their integration with electronic neural networks. *IEEE J. Sel. Top. Quantum Electronics* **26**, 3700114 (2019).

7. Nanoscribe. https://www.nanoscribe.com/en/.

8. Turner, M. D. *et al.,* Miniature chiral beamsplitter based on gyroid photonic crystals. *Nature Photonics* **7**, 801–807 (2013).

9. Goi, E., Cumming, B. P. & Gu, M., Impact of cubic symmetry on optical activity of dielectric 8-srs networks. *Applied Sciences* **8**, 2104 (2018).

10. Bückmann, T. *et al.,* Tailored 3D mechanical metamaterials made by dip-in direct-laser-writing optical lithography. *Advanced Materials* **24**, 2710–2714 (2012).

11. Shen, Y., *et al.*, M. Deep learning with coherent nanophotonic circuits. *Nature Photonics* **11**, 441–447 (2017).

12. Vivien, L. *et al.,* Zero-bias 40Gbit/s germanium waveguide photodetector on silicon. *Optics Express* **20**, 1096–1101 (2012).

13. Shen, Y. *et al.,* Deep learning with coherent nanophotonic circuits. *Nature Photonics* **11**, 441–447 (2017).
